# Targeting Hypoxia and Autophagy Inhibition via Delivering Sonodynamic Nanoparticles With HIF‐2α Inhibitor for Enhancing Immunotherapy in Renal Cell Carcinoma

**DOI:** 10.1002/adhm.202402973

**Published:** 2024-10-13

**Authors:** Yihao Zhu, Yajian Li, Xuwen Li, Yuan Yu, Lingpu Zhang, Hanchen Zhang, Can Chen, Dong Chen, Mingshuai Wang, Nianzeng Xing, Feiya Yang, Wahafu Wasilijiang, Xiongjun Ye

**Affiliations:** ^1^ Department of Urology National Cancer Center/National Clinical Research Center for Cancer/Cancer Hospital Chinese Academy of Medical Sciences and Peking Union Medical College Beijing 100021 China; ^2^ Zhejiang Cancer Hospital Hangzhou Institute of Medicine Chinese Academy of Sciences Zhejiang 310022 China; ^3^ Beijing National Laboratory for Molecular Sciences Laboratory of Polymer Physics and Chemistry Institute of Chemistry Chinese Academy of Sciences Beijing 100190 China; ^4^ University of Chinese Academy of Sciences Beijing 100049 China; ^5^ Department of Oncology The Second Affiliated Hospital of Zunyi Medical University Guizhou 563000 China; ^6^ Department of Urology Shanxi Province Cancer Hospital/Shanxi Hospital Affiliated to Cancer Hospital Chinese Academy of Medical Sciences/Cancer Hospital Affiliated to Shanxi Medical University Shanxi 030013 China

**Keywords:** HIF‐2α, immunotherapy, renal cell carcinoma, sonodynamic therapy

## Abstract

Immune checkpoint blockers (ICBs) therapy stands as the first‐line treatment option for advanced renal cell carcinoma (RCC). However, its effectiveness is hindered by the immunosuppressive tumor microenvironment (TME). Sonodynamic therapy (SDT) generates tumor cell fragments that can prime the host's antitumor immunity. Nevertheless, the hypoxic microenvironment and upregulated autophagy following SDT often lead to cancer cell resistance. In response to these challenges, a hypoxia‐responsive polymer (Poly(4,4′‐azobisbenzenemethanol‐PMDA)‐mPEG_5k_, P‐APm) encapsulating both a HIF‐2α inhibitor (belzutifan) and the ultrasonic sensitize (Chlorin e6, Ce6) is designed, to create the nanoparticle APm/Ce6/HIF. APm/Ce6/HIF combined with ultrasound (US) significantly suppresses tumor growth and activates antitumor immunity in vivo. Moreover, this treatment effectively transforms the immunosuppressive microenvironment from “immune‐cold” to “immune‐hot”, thereby enhancing the response to ICBs therapy. The findings indicate that APm/Ce6/HIF offers a synergistic approach combining targeted therapy with immunotherapy, providing new possibilities for treating RCC.

## Graphical abstract

This study reports on a hypoxia‐responsive nanoparticle loaded with a HIF‐2α inhibitor (belzutifan), and the ultrasonic sensitizer (Chlorin e6, Ce6), which can inhibit tumor hypoxia and autophagy signaling pathways, and downregulate PD‐L1 expression in tumor cells, leading to improved efficacy of αPD‐1. Overall, it presents a promising strategy of targeted therapy combined with immunotherapy for treating renal cell carcinoma.

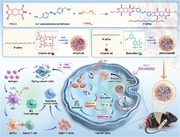

## 1 Introduction

In the urinary system, renal cell carcinoma (RCC) is one of the most common types of malignant tumor.^[^ ^1^ ^]^ At the time of initial diagnosis, ≈30% of kidney cancer patients have distant metastases.^[^ ^2^ ^]^ The prognosis of advanced RCC patients is poor, with a 5‐year survival rate of only ≈35–40%.^[^ ^3^ ^]^ Immunotherapy based on immune checkpoint blockers (ICBs) is the first‐line treatment for patients with advanced kidney cancer.^[^ ^4^ ^]^ However, the response rates for immunotherapy‐treated patients with advanced kidney cancer range from 33% to 60%, and only 3–11% achieve complete response rates.^[^ ^5^ ^]^ The low response rate to ICBs is primarily caused by the immunosuppressive tumor microenvironment (TME), which suppresses antitumor immunity and hampers the effectiveness of immunotherapy.^[^ ^6^ ^]^ Therefore, the development of novel strategies to counter tumor immunosuppression and enhance the efficacy of immunotherapy is highly significant.

In recent years, sonodynamic therapy (SDT) has garnered significant attention as a potential cancer treatment modality. Compared to photodynamic therapy (PDT), SDT has several advantages, such as improved tissue penetration, high long‐range spatiotemporal selectivity, and non‐invasive treatment.^[^ ^7^ ^]^ Recently, it has been reported that tumor cell fragments generated during SDT can serve as a source of tumor antigens and stimulate antitumor immunity.^[^ ^8^ ^]^ Studies have shown that during SDT, ultrasonic sensitizers absorb ultrasonic energy and convert oxygen into reactive oxygen species (ROS), leading to increased oxygen consumption and exacerbating the hypoxic state of the TME, thereby limiting the effectiveness of SDT.^[^ ^9^ ^]^ Furthermore, studies have shown that a hypoxic TME can inhibit the efficacy of immunotherapy.^[^ ^10^ ^]^ In this context, altering tumor hypoxia by inhibiting the hypoxia signaling pathway may improve the therapeutic effect of ICBs.

Belzutifan is a next‐generation targeted therapeutic that has been approved for the treatment of RCC, central nervous system hemangioblastoma (CNS), and pancreatic neuroendocrine tumors (pNET) associated with von Hippel‐Lindau (VHL) syndrome.^[^ ^11^ ^]^ It exerts its function by inhibiting the expression of HIF‐2α and the hypoxia signaling pathway.^[^ ^12^ ^]^ Compared with HIF‐1, HIF‐2 offers better tissue and cell specificity and is believed to have an indispensable role in various tumors.^[^ ^13^ ^]^ The regulation of HIF‐2 primarily relies on the prolyl hydroxylase domain enzyme (PHDs), and its degradation is facilitated by the ubiquitin‐proteasome system mediated by the VHL protein. Studies have shown that long noncoding RNA‐SARCC could inhibit the proliferation of VHL mutant renal cancer cells and promote the proliferation of VHL normal renal cancer cells by regulating the androgen receptor /HIF‐2α/C‐MYC axis under the hypoxic microenvironment.^[^ ^14^ ^]^ Furthermore, studies have indicated that HIF‐2α is critical for the function of regulatory T cells, and targeting HIF‐2α shows potential for effectively inhibiting tumor growth.^[^ ^15^ ^]^

**Figure 1:**
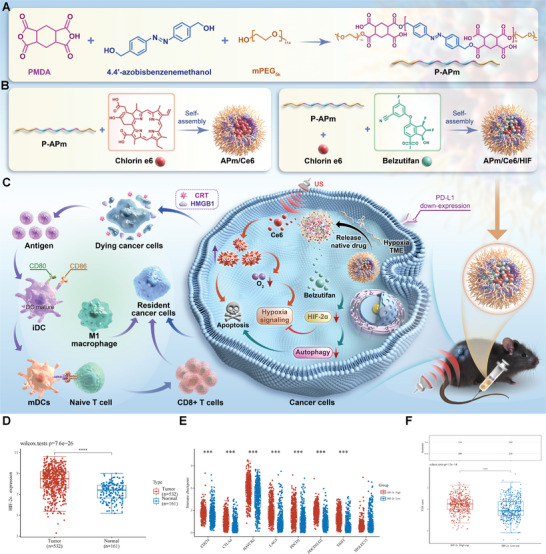
Belzutifan sensitizes tumor cells to SDT via inhibition of HIF‐2α which correlates with the treatment outcome in RCC patients. A) Synthesis of P‐APm. B) The preparation of APm/Ce6 and APm/Ce6/HIF. C) Schematic illustration of APm/Ce6/HIF therapy in vivo. Initially, APm/Ce6/HIF is taken up by renal carcinoma cells. Subsequently, APm/Ce6/HIF rapidly degrade in the hypoxic TME, releasing belzutifan and Ce6. The released belzutifan effectively suppresses tumor growth by targeting hypoxia and autophagy signaling pathways. Furthermore, Ce6 enhances SDT, significantly increasing the generation of ROS and resulting in the release of damage‐associated molecular patterns (DAMPs), such as calreticulin (CRT) and high mobility group box 1 (HMGB1), thereby inducing dendritic cells maturation, promoting the T‐cell infiltration, increasing the transformation of M2 macrophages to M1 macrophages in the tumor immune microenvironment and decreasing the programmed cell death protein ligand 1 (PD‐L1) expression. Thus, the response rates of APm/Ce6/HIF+US combined αPD‐1 improve. D) The expression of HIF‐2α in 532 renal tumors from the TCGA dataset and 161 normal renal tissues from the GTEx dataset were analyzed via the ACLBI website. E) The expression distribution of immune checkpoints gene in tumor tissues and normal tissues. The abscissa represents different groups of samples, and the ordinate represents the expression distribution of genes, different colors represent different groups. F) Prediction of potential immunotherapy response using the TIDE algorithm. Top: Statistical table of immune responses in different groups of predicted results; bottom: Distribution of immune response scores in different groups of predicted results, where different colors represent expression trends in different samples. The significance of two groups of samples is tested by the Wilcoxon test. ***p < 0.001, ****p < 0.0001.

In this study, we designed a hypoxia‐responsive polymer (Poly(4,4′‐azobisbenzenemethanol‐PMDA)‐mPEG_5k_, P‐APm) loaded with a HIF‐2α inhibitor (belzutifan), and the ultrasonic sensitizer (Chlorin e6, Ce6), forming the nanoparticle APm/Ce6/HIF (**Figure** **1**A,B). APm/Ce6/HIF could accumulate in RCC tumor‐bearing mice effectively in tumor sites after systemic administration. In the hypoxic TME, APm/Ce6/HIF rapidly degraded, releasing the encapsulated Ce6 and belzutifan (Figure S1A,B, Supporting Information). On the one hand, APm/Ce6/HIF combined with ultrasound (US) could achieve the SDT effect by producing ROS to kill cancer cells. On the other hand, belzutifan could produce synergistic antitumor effects by inhibiting tumor hypoxia and autophagy signaling pathways. Moreover, the presence of APm/Ce6/HIF+US altered the immunosuppressive TME and transformed the “immune‐cold” tumor into an “immune‐hot” tumor, leading to improved efficacy of the anti‐programmed cell death protein‐1 antibody (αPD‐1). Overall, this study presents a promising strategy of targeted therapy combined with immunotherapy for treating RCC, demonstrating significant clinical application potential (Figure 1C).

## 2 Results

### 2.1 HIF‐2α Is Highly Expressed in RCC and Is Associated With Poor Immunotherapeutic Efficacy

HIF‐2α plays a pivotal role in the hypoxia signaling pathway. Previous studies have indicated that in the hypoxic microenvironment, pancreatic ductal adenocarcinoma (PDAC) cells expressing activated *KRAS* can upregulate the expression of CA9 by stabilizing HIF‐1α and HIF‐2α, resulting in drug resistance to gemcitabine.^[^ ^16^ ^]^ To investigate the role of HIF‐2α in RCC, we obtained RNA sequencing (RNA‐Seq) data of RCC tissues and normal renal tissues from the Cancer Genome Atlas (TCGA) dataset and Genotype‐Tissue Expression (GTEx) dataset. As shown in Figure 1D, the expression of HIF‐2α in RCC tissues was significantly higher than in normal renal tissues, suggesting its association with RCC progression. Immune checkpoint molecules are important regulatory molecules that inhibit immune cell functions and antitumor immunity, leading to tumor progression and immune escape. To investigate the role of HIF‐2α in antitumor immunity in RCC, we initially analyzed its relationship with immune checkpoint molecules using the TCGA database. The expression of immunosuppression‐related genes, such as CD274 and cytotoxic T lymphocyte‐associated protein 4 (CTLA4), in patients with high HIF‐2α expression were higher than those in patients with low HIF‐2α expression (Figure 1E). Furthermore, based on the expression profile data, the Tumor Immune Dysfunction and Exclusion (TIDE) algorithm was used to predict the relationship between HIF‐2α expression and RCC patient response to immune checkpoint inhibitors (ICIs). As shown in Figure 1F, patients with high HIF‐2α expression exhibited a poor response to ICIs compared to patients with low HIF‐2α expression. Collectively, these findings suggest that overexpression of HIF‐2α in RCC may contribute to suboptimal clinical responses to immunotherapy, with inhibiting HIF‐2α expression representing a promising strategy to enhance the efficacy of immunotherapy in RCC patients.

### 2.2 Belzutifan Inhibited the Proliferation of HIF‐2α‐Expressing RCC Cells in the Environment

To investigate the role of HIF‐2α in RCC, the expression of HIF‐2α was measured in various RCC cells, including A498 (human renal cell carcinoma cell), ACHN (human renal adenocarcinoma cell), and 786‐O (human renal adenocarcinoma cell). It was found that HIF‐2α was expressed in A498 and 786‐O. The expression of HIF‐2α was increased in A498 and 786‐O in the hypoxic environment (Figure S2A, Supporting Information), suggesting that HIF‐2α could potentially support the proliferation of RCC cells in the hypoxic environment. To further investigate the role of HIF‐2α, belzutifan was added to inhibit the expression of HIF‐2α. In the hypoxic environment, the addition of belzutifan resulted in significant inhibition of proliferation in 786‐O and A498 cells, while no inhibition was observed in ACHN cells (Figure S2B–D, Supporting Information). At belzutifan concentrations of 41.56 and 57.87 µm, the cell viability of 786‐O and A498 cells respectively decreased to 50%. To validate these results further, we conducted CCK8 experiments. In the hypoxic environment, the inhibitory effect of belzutifan led to minimal proliferation in 786‐O and A498 cells within 72 h, whereas the cell proliferation of ACHN was not significantly affected by belzutifan (Figure S2E–G, Supporting Information). These results showed that belzutifan effectively reduces the proliferation of HIF‐2α‐expressing RCC cells in the hypoxic environment.

### 2.3 Preparation, Characterization, and Cellular Uptake of APm/Ce6 and APm/Ce6/HIF

**Figure 2:**
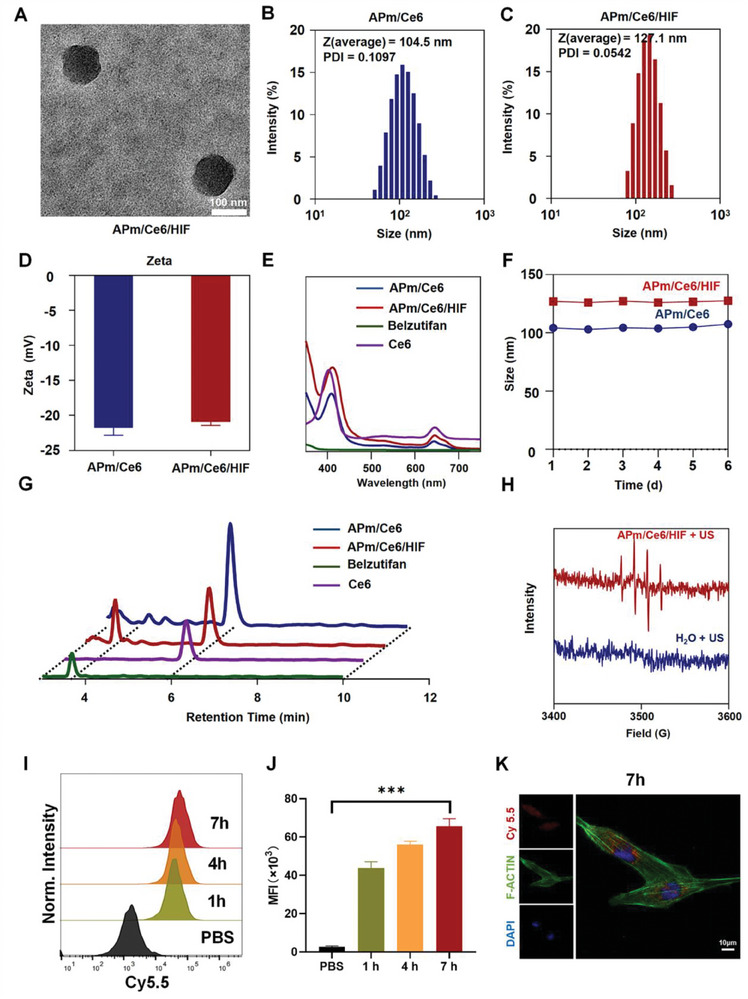
Characterization and cell uptake of APm/Ce6 and APm/Ce6/HIF. A) TEM was performed to characterize the APm/Ce6/HIF. Scale bar: 100 nm. B‐D) DLS was performed to measure the particle size and potential of APm/Ce6 and APm/Ce6/HIF (D: n = 3). E) UV absorption of APm/Ce6 and APm/Ce6/HIF. F) DLS was used to detect the particle size of APm/Ce6 and APm/Ce6/HIF at different time points. G) HPLC was used to analyze the release of APm/Ce6 and APm/Ce6/HIF in the presence of Na_2_S_2_O_4_. H) ESR was used to verify the generation of hydroxyl radicals by APm/Ce6/HIF under the action of ultrasound. I–J) FCM was used to verify the cell uptake of APm/Cy5.5 at different time points (J: n = 3). K) CLSM was used to visualize the cell uptake of APm/Cy5.5 at 7 h. Scale bar: 10 µm. Data are presented as mean ± SD. Statistical significance was calculated by one‐way analysis of variance. ***p < 0.001.

The hypoxic microenvironment of solid tumors compared with normal tissues has been confirmed by many researchers.^[^ ^17^ ^]^ To prepare hypoxia‐responsive nanoparticles loaded with Ce6 and belzutifan for further enhancing their synergistic effect, herein, we synthesized 4,4′‐azobisbenzenemethanol using 4‐nitrobenzyl alcohol as a hypoxia‐responsive linker (Scheme S1, Supporting Information), which was confirmed by ^1^H‐NMR (Figure S3, Supporting Information). Subsequently, to obtain hypoxia‐responsive prodrugs (P‐APm), the polymer was synthesized via 1,2,4,5‐cyclohexanetetracarboxylic dianhydride (PMDA), 4,4′‐azobisbenzenemethanol and polyethylene glycol monomethyl ether (mPEG_5k_) (Figure 1A). The successful synthesis of P‐APm was also confirmed by ^1^H‐NMR (Figure S4, Supporting Information). Then, the hypoxia‐responsive nanoparticles (APm/Ce6/HIF) were prepared by encapsulating Ce6 and belzutifan simultaneously with P‐APm through self‐assembly. Additionally, nanoparticles (APm/Ce6) encapsulated with Ce6 only were prepared using the same method. The physicochemical characteristics of APm/Ce6 and APm/Ce6/HIF were evaluated. Transmission electron microscopy (TEM) revealed that APm/Ce6 and APm/Ce6/HIF have a uniform, spherical shape with an approximate diameter of 100 and 120 nm, respectively. (Figure S5, Supporting Information; **Figure** **2**A). Dynamic light scattering (DLS) analysis confirmed that the average particle size of APm/Ce6 and APm/Ce6/HIF was 104.5 nm (Figure 2B) and 127.1 nm (Figure 2C) respectively, and the zeta potential of APm/Ce6 and APm/Ce6/HIF was −21.8 and −20.9 mV (Figure 2D) respectively. As shown in Figure 2E, UV absorption spectra of APm/Ce6 and APm/Ce6/HIF both indicated the characteristic absorption peak of Ce6, indicating that both nanoparticles contained Ce6. Furthermore, the stability of APm/Ce6 and APm/Ce6/HIF under physiological conditions was assessed by monitoring their particle sizes in phosphate buffer saline (PBS, pH 7.4) over 6 consecutive days. The average size of both APm/Ce6 and APm/Ce6/HIF remained unchanged in PBS, demonstrating their superior stability (Figure 2F).

To examine whether APm/Ce6 and APm/Ce6/HIF could respond to hypoxic environments, Na_2_S_2_O_4_ was used to simulate the hypoxic environments as previously described.^[^ ^18^ ^]^ When APm/Ce6 and APm/Ce6/HIF were incubated with Na_2_S_2_O_4_ for 1 h, we observed the release of Ce6 from APm/Ce6 and the release of Ce6 and belzutifan from APm/Ce6/HIF, as confirmed by high‐performance liquid chromatography (HPLC) analysis (Figure 2G). This finding indicates that both APm/Ce6 and APm/Ce6/HIF are responsive to hypoxia and can effectively release drugs in the hypoxic environment. Furthermore, the ability of APm/Ce6/HIF to generate ROS under ultrasonic conditions (1 W cm^−2^, frequency of 1 MHz, duty ratio of 50%, 2 min) was confirmed by electron spin resonance (ESR) spectrometer. The ROS levels in water and the solutions of APm/Ce6/HIF before and after ultrasonic treatment were measured by ESR spectrometer, showing that APm/Ce6/HIF could generate ROS under the selected conditions (Figure 2H).

To assess the cellular uptake of APm/Ce6/HIF, 786‐O cells were treated with Cy5.5 labeled APm/Ce6/HIF (APm/Cy5.5) for different durations. It was found that the mean fluorescence intensity (MFI) was 1.50 times following 7 h of treatment compared with that at 1 h by Flow cytometric (FCM) (Figure 2I,J, Supporting Information). Moreover, the uptake of APm/Cy5.5 by 786‐O cells was further confirmed using confocal laser scanning microscopy (CLSM). Fluorescence signals (red) were observed inside the cells 7 h after incubation with APm/Cy5.5 (Figure 2K). These results indicate that APm/Ce6/HIF can be effectively taken up by cancer cells and can release belzutifan and Ce6 in the hypoxic environment.

### 2.4 The Anticancer Activity of APm/Ce6/HIF In Vitro

**Figure 3:**
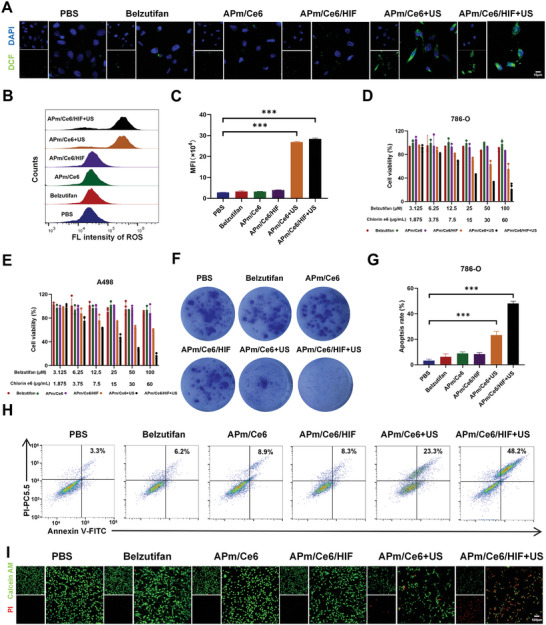
The antitumor activity of APm/Ce6/HIF in vitro. A) Intracellular ROS generation of 786‐O cells treated with various treatments by CLSM. B, C) ROS generation of 786‐O cells treated with various treatments and the corresponding quantification of ROS generation by FCM (C: n = 3). D, E) In vitro cytotoxicity of different treatments on 786‐O and A498 cells for 24 h (n = 3). F) Colony formation assay was used to detect the effect of different treatments on colony formation of 786‐O cells. G, H) Apoptosis rate of 786‐O cells treated with various treatments by FCM (G: n = 3). I) Live and dead cells of 786‐O cells stained with Calcein‐AM (green, alive) and PI (red, dead) were analyzed by CLSM. Scale bar: 100 µm. Data are presented as mean ± SD. Statistical significance was calculated by one‐way analysis of variance. ***p < 0.001.

Considering that APm/Ce6/HIF could generate singlet oxygen under US conditions, we first investigated the capability of APm/Ce6/HIF to generate intracellular ROS. DCFH‐DA (2′,7′‐dichlorodihydrofluorescein diacetate) was used as a probe for intracellular ROS, which can be oxidized to produce fluorescent DCF in the presence of ROS. The results by CLSM showed that there was green fluorescence in cells treated with both APm/Ce6+US and APm/Ce6/HIF+US (exposed to ultrasound (1 W cm^−2^, frequency of 1 MHz, duty ratio of 50%) for 2 min), indicating that more ROS was generated (**Figure** **3**A). What's more, a semiquantitative analysis by FCM for the ROS generation in cells treated with various treatments was performed. The results showed that the intracellular green fluorescence of cells treated with APm/Ce6+US and APm/Ce6/HIF+US was 9.6‐ and 10.1‐fold higher than that of cells treated with PBS, respectively (Figure 3B,C). The above results together indicated that both APm/Ce6+US and APm/Ce6/HIF+US could generate ROS efficiently.

To evaluate the in vitro anticancer efficacy of APm/Ce6/HIF, 786‐O cells were treated with belzutifan, APm/Ce6, APm/Ce6/HIF, APm/Ce6+US and APm/Ce6/HIF+US for 24 h, and the cell proliferation was measured using the MTT assay. The results showed that APm/Ce6+US significantly reduced the cell viability to 54.1% due to the cytotoxic ROS generated by APm/Ce6+US. Moreover, APm/Ce6/HIF+US reduced the cell viability to 21.7%, indicating the synergistic effect of belzutifan with APm/Ce6+US (Figure 3D). Similar results were also found in A498 cells (Figure 3E), with APm/Ce6/HIF+US exhibiting the highest anticancer efficacy. The colony formation assay was also used to evaluate the anticancer efficacy of APm/Ce6/HIF+US. The results showed that APm/Ce6/HIF+US significantly inhibited the colony formation of 786‐O cells (Figure 3F). Furthermore, the apoptosis rate of 786‐O cells treated with various treatments was examined by an Annexin V‐FITC and propidium iodide (PI) double‐staining assay, revealing that the apoptosis rate induced by APm/Ce6/HIF+US was the highest (48.2%) among all treatments, significantly higher than that induced by APm/Ce6+US (23.3%) (Figure 3G,H). Moreover, Live/Dead staining indicated that APm/Ce6/HIF+US induced more dead cells than other treatments (Figure 3I).

### 2.5 APm/Ce6/HIF+US Inhibit the Proliferation of Renal Carcinoma Cells by Inhibiting Hypoxia and Autophagy Signaling Pathways

**Figure 4:**
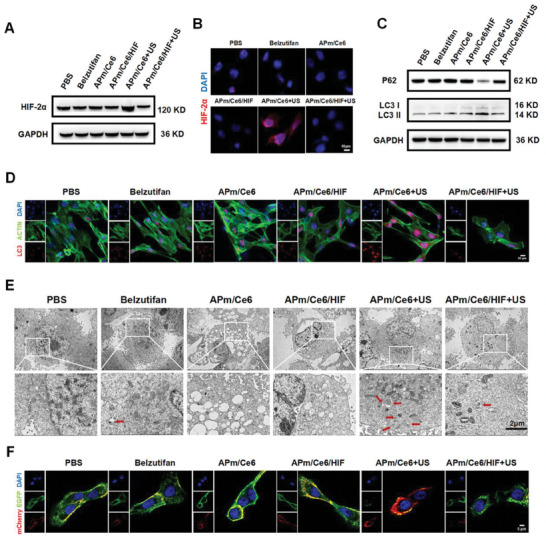
APm/Ce6/HIF+US inhibited the proliferation of renal cancer cells by inhibiting hypoxia and autophagy signaling pathways. A) The expression of HIF‐2α in 786‐O cells treated with various treatments by western blot. B) The expression of HIF‐2α in 786‐O cells treated with various treatments by CLSM (red: HIF‐2α, blue: DAPI). Scale bar: 10 µm. C) The expression of P62 and LC3 I/II in 786‐O cells treated with various treatments by western blot. D) The expression of LC3 in 786‐O cells treated with various treatments by CLSM (red: LC3, green: ACTIN, blue: DAPI). Scale bar: 10 µm. E) The autophagosome and autophagolysosomes of 786‐O cells treated with various treatments by TEM (red arrows refer to autophagosome and autophagolysosomes). Scale bar: 2 µm. F) Intracellular autophagy of 786‐O cells treated with various treatments by CLSM. Scale bar: 5 µm.

Previous studies have revealed that SDT can worsen the hypoxic TME and stimulate autophagy in cancer cells.^[^ ^19^ ^]^ Additionally, studies have also shown that increased expression of HIF‐2α in cancer cells promotes autophagy.^[^ ^20^ ^]^ To investigate whether APm/Ce6+US could induce the expression of HIF‐2α in renal cancer cells and promote autophagy, the expression of HIF‐2α in 786‐O cells treated with various treatments was examined by western blot. Compared to the other treated cells, it was found that the expression of HIF‐2α in 786‐O cells treated with APm/Ce6+US was significantly increased, indicating that APm/Ce6+US could lead to a hypoxic TME. In contrast, the expression of HIF‐2α was decreased in 786‐O cells treated with APm/Ce6/HIF+US compared with that in cells treated with APm/Ce6+US (**Figure** **4**A). Then the semiquantitative analysis by FCM showed that the intracellular red fluorescence of cells treated with APm/Ce6+US was 1.6‐fold higher than that of cells treated with PBS, and the intracellular red fluorescence of cells treated with APm/Ce6/HIF+US was 0.4‐fold lower than that of cells treated with APm/Ce6+US, proving that belzutifan in APm/Ce6/HIF could inhibit the expression of HIF‐2α (Figure 4B; Figure S6A, Supporting Information).

LC3 is a core autophagy‐related protein, and when autophagy is formed, the cytoplasmic form of LC3 (LC3 I) enzymatically cleaves a small polypeptide and converts to the membranous form (LC3 II).^[^ ^21^ ^]^ To explore the occurrence of autophagy in cancer cells treated with various treatments, it was found that the expression of LC3 II was significantly increased in 786‐O cells treated with APm/Ce6+US compared with that in cells treated with APm/Ce6/HIF+US by western blot. Meanwhile, the common endogenous autophagy substrate SQSTM1/P62 was significantly decreased in 786‐O cells treated with APm/Ce6+US (Figure 4C). The expression of intracellular LC3 was further analyzed by immunofluorescence staining. The intracellular red fluorescence of cells treated with APm/Ce6+US was 2.2‐fold higher than that of cells treated with PBS, proving that the expression of LC3 was significantly increased after APm/Ce6+US treatment (Figure 4D; Figure S6B, Supporting Information). Since the presence of autophagosomes and autophagolysosomes is considered the gold standard for detecting autophagy, their presence was observed using TEM. The results showed that the number of autophagosomes and autophagolysosomes in 786‐O cells treated with APm/Ce6+US was highest (Figure 4E), suggesting that belzutifan could inhibit the proliferation of renal cancer cells by inhibiting autophagy signaling pathway. To further verify the occurrence of autophagy in 786‐O cells treated with various treatments, 786‐O cells that stably expressed mCherry‐EGFP‐LC3B were constructed. In the absence of autophagy, mCherry‐EGFP‐LC3B exists in the cytoplasm as diffuse yellow fluorescence (the combined effect of mCherry and EGFP) under fluorescence microscopy. However, when autophagy is activated, mCherry‐EGFP‐LC3B aggregates on the autophagosome membrane, appearing as yellow spots under fluorescence microscopy. Subsequently, when the autophagosome fuses with the lysosome, the acidic environment of the autophagosome quenches the EGFP fluorescence, resulting in the appearance of red spots. As shown in Figure 4F, there was more intracellular red fluorescence in 786‐O cells treated with APm/Ce6+US. These results indicated that APm/Ce6+US promoted autophagy in 786‐O cells, which could be inhibited by belzutifan. In summary, APm/Ce6/HIF+US showed the highest anticancer efficacy in RCC through inhibiting hypoxia and autophagy signaling pathways.

### 2.6 APm/Ce6/HIF+US Induced Immunogenic Cell Death (ICD) of RCC In Vitro

**Figure 5:**
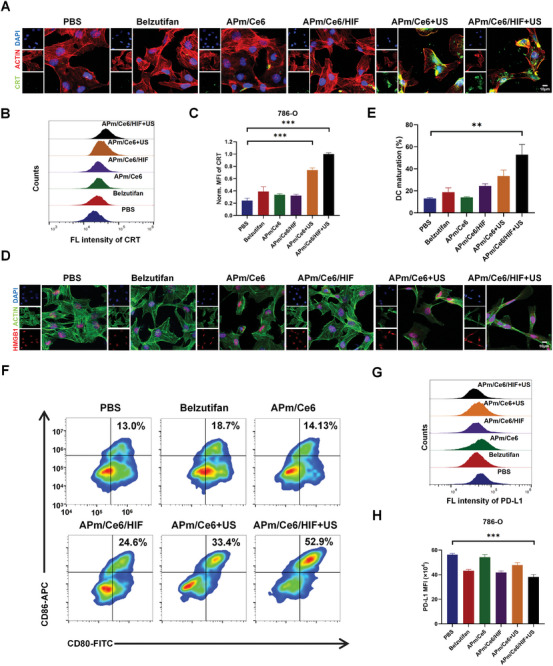
APm/Ce6/HIF+US induced immunogenic cell death of renal cancer cells in vitro. A) The expression of CRT in 786‐O cells treated with various treatments by CLSM (CRT: green, ACTIN: red, DAPI: blue). Scale bar: 10 µm. B,C) The expression of CRT in 786‐O cells treated with various treatments by FCM (C: n = 3). D) The expression of HMGB1 in 786‐O cells treated with various treatments by CLSM (HMGB1: red, ACTIN: green, DAPI: blue). Scale bar: 10 µm. E,F) CD11C, CD80, and CD86 expression on BMDCs after various treatments (E: n = 3). G,H) The expression of PD‐L1 in 786‐O cells treated with various treatments by FCM (H: n = 3). Data are presented as mean ± SD. Statistical significance was calculated by one‐way analysis of variance. **p < 0.01, ***p < 0.001.

ICD refers to the process of cancer cells transitioning from non‐immunogenic to immunogenic mediated anticancer immune response when they die due to external stimulation. Previous studies have shown that ROS can induce ICD and release a series of DAMPs. During ICD, cancer cells produce various signaling molecules, including CRT exposed on the cell surface, HMGB1, ATP molecules, and heat shock proteins (HSP70, HSP90).^[^ ^22^ ^]^ To verify whether APm/Ce6/HIF+US inhibited the proliferation of renal cancer cells via ICD, the expression of CRT on the cell surface in 786‐O cells was measured by immunofluorescence staining. As shown in **Figure** **5**A, the expression of CRT on the surface of 786‐O cells treated with APm/Ce6+US and APm/Ce6/HIF+US was significantly increased. What's more, a semiquantitative analysis by FCM for the expression of CRT in cells treated with various treatments was performed. The results showed that the MFI of CRT in 786‐O cells treated with APm/Ce6+US and APm/Ce6/HIF+US was 3.0‐ and 4.1‐fold higher than that of cells treated with PBS, respectively (Figure 5B,C). Subsequently, the expression and localization of HMGB1 in 786‐O cells were examined. The results showed that the expression of HMGB1 in 786‐O cells treated with APm/Ce6+US and APm/Ce6/HIF+US was increased, and it migrated from the nucleus to the cytoplasm (Figure 5D). Studies have shown that DAMPs released during ICD can bind to Pattern Recognition Receptors (PRRs) on the surface of dendritic cells (DCs), initiating a series of cellular reactions that activate innate and adaptive immune responses^[^ ^22^ ^]^ DCs are important antigen‐presenting cells affecting the activation of innate and adaptive immunity. Accordingly, the ability of APm/Ce6/HIF+US to activate immune responses was further investigated in vitro. RENCA (Murine‐derived renal carcinoma cells) cells were pretreated with various treatments. Bone‐marrow‐derived dendritic cells (BMDCs) isolated from C57BL/6 were then co‐incubated with pretreated RENCA cells. As shown in Figure 5E,F, APm/Ce6/HIF+US triggered a significant upregulation of CD80^+^ and CD86^+^ (52.9%) in BMDCs, which was 4.1 and 1.6 times higher than those treated with PBS (13.0%) and APm/Ce6+US (33.4%) respectively. These results suggested that APm/Ce6/HIF+US could induce DCs maturation through ICD and stimulate anticancer immunity.

Immunotherapy targeting the programmed cell death protein 1 (PD‐1) and PD‐L1 has recently emerged as a crucial approach to tumor treatment. PD‐L1 is a ligand‐protein produced by cancer cells that binds to PD‐1 on T lymphocytes, transmitting immunosuppressive signals that reduce the activation and proliferation of CD8^+^ T cells in lymph nodes. Previous studies have shown that HIF‐1α can promote the expression of PD‐L1 in cancer cells.^[^ ^23^ ^]^ To investigate the relationship between the expression of HIF‐2α and PD‐L1 on the cell surface, it was detected that APm/Ce6/HIF+US could reduce the expression of PD‐L1 on the surface of 786‐O cells by FCM (Figure 5G,H). In summary, APm/Ce6/HIF+US not only induced ICD but also decreased the expression of PD‐L1 on the surface of 786‐O cells.

### 2.7 Biodistribution and Antitumor Efficacy of APm/Ce6/HIF In Vivo

**Figure 6:**
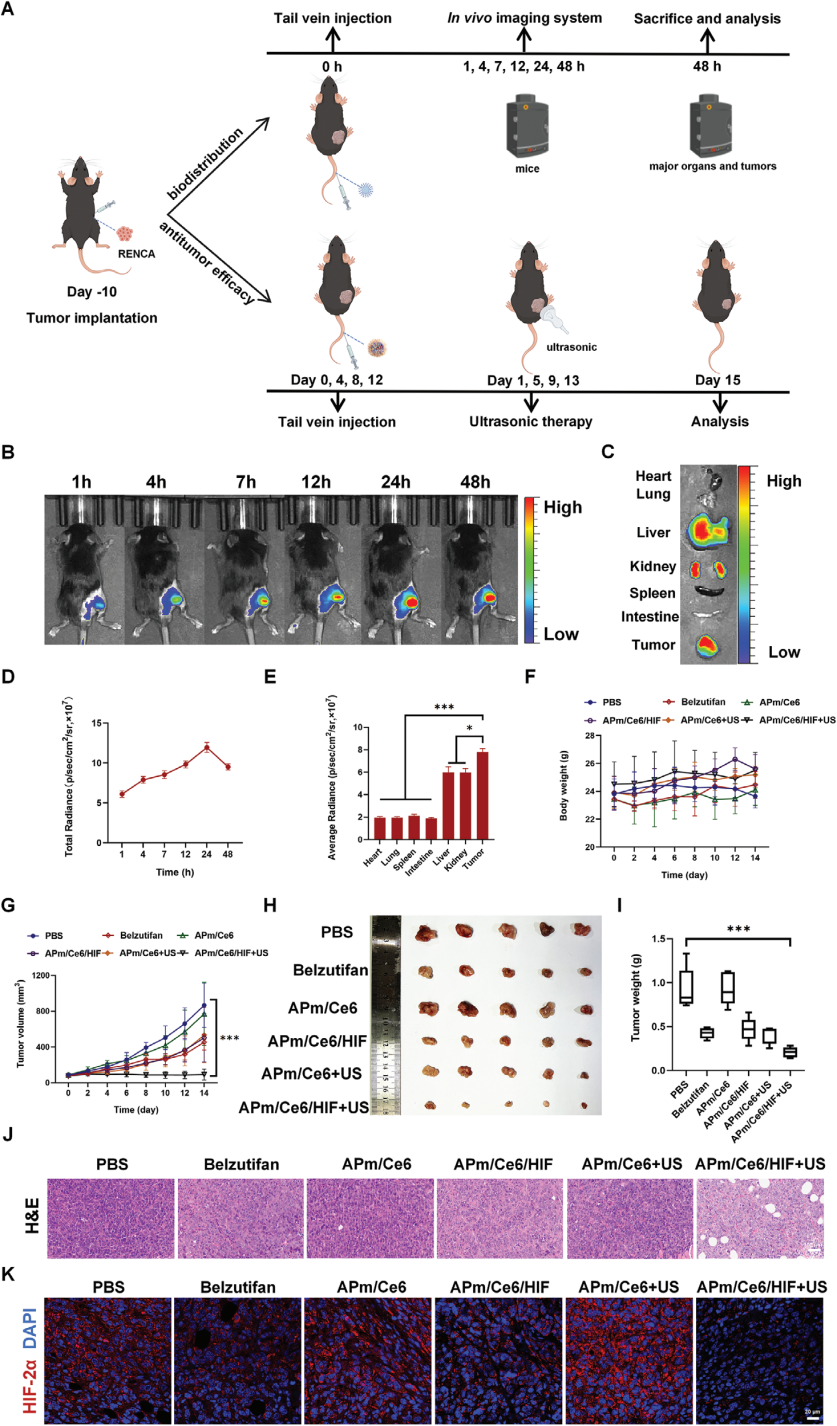
Biodistribution and antitumor efficacy of APm/Ce6/HIF in vivo. A) Schematic illustration of tumor model establishment, biodistribution, and treatment scheme. B) Quantification of fluorescence intensity of tumor after intravenous injection of APm/Cy7.5 at different times (IVIS, spectral CT, PerkinElmer, Ex/Em = 745 nm/840 nm). C) Fluorescence images of major organs 48 h after intravenous injection of APm/Cy7.5 by IVIS. D) Quantification of fluorescence intensity of tumor after intravenous injection of APm/Cy7.5 at different times (n = 5, IVIS, spectral CT, PerkinElmer, Ex/Em = 745 nm/840 nm). E) Mean fluorescence intensity of organs after 48 h of intravenous injection of APm/Cy7.5 by IVIS (n = 5). F) The weight of mice treated with PBS, belzutifan, APm/Ce6, APm/Ce6/HIF, APm/Ce6+US and APm/Ce6/HIF+US (n = 5). G) Curve of tumor growth of mice treated with various treatments (n = 5). H, I) Tumor images and corresponding tumor weight of mice treated with various treatments (n = 5). J) H&E staining of tumor tissue. Scale bar: 100 µm. K) Immunofluorescence analysis of HIF‐2α expression in cells of tumor tissue of mice treated with various treatments by CLSM. Scale bar: 20 µm. Data are presented as mean ± SD. Statistical significance was calculated by one‐way analysis of variance. *p < 0.05, ***p < 0.001.

Optimal biodistribution of nanoparticles in vivo is the basis of therapeutic efficacy. In this study, we investigated the biodistribution of Cy7.5‐labelled APm/Ce6/HIF (APm/Cy7.5) by an in vivo imaging system (IVIS) and in vivo antitumor efficacy of APm/Ce6/HIF in RENCA tumor‐bearing mice (**Figure** **6**A). After systemic injection, the signals of APm/Cy7.5 at the tumor site gradually increased and reached their maximum at 24 h post‐injection (Figure 6B). At 48 h after injection, the mice were sacrificed, and IVIS was used to detect the fluorescence signals of APm/Cy7.5 in major organs and tumors. The results showed that APm/Cy7.5 primarily accumulated in the tumors, with intense red fluorescence also observed in the liver and kidney (Figure 6C). What's more, a semiquantitative analysis by IVIS for the fluorescence signal indicated that the tumor site gradually increased and reached its maximum at 24 h, with a peak of 1.195 × 10^8^ p s^−1^ cm^−2^ S^−1^ r^−1^. Notably, a strong fluorescence signal could still be detected at 48 h post‐injection (9.520 × 10^7^ p s^−1^ cm^−2^ S^−1^ r^−1^), indicating that APm/Cy7.5 had superior tumor accumulation ability (Figure 6D). The fluorescence intensity in tumors remained 3.99, 3.99, 3.70, 1.30, and 1.31 times higher than those in the heart, lung, spleen, intestine, liver, and kidney at 48 h, respectively (Figure 6E). The above results proved that APm/Ce6/HIF could effectively accumulate in tumors after systemic injection.

After exploring the biodistribution of APm/Ce6/HIF, the in vivo antitumor efficacy of APm/Ce6/HIF was investigated. The therapeutic efficacy of APm/Ce6/HIF+US in RENCA tumor‐bearing mice was evaluated (Figure 6A). When the subcutaneous tumors reached 100 mm^3^, the mice were treated with a single dose of PBS, belzutifan (5 mg kg^−1^, intragastric gavage), APm/Ce6 (3 mg kg^−1^ Ce6, intravenous injections) and APm/Ce6/HIF (3 mg kg^−1^ Ce6, 5 mg kg^−1^ belzutifan, intravenous injections) respectively. Then, the APm/Ce6+US and APm/Ce6/HIF+US treated mice were treated with ultrasound for 3 min on the 2nd day of drug injection. The RENCA tumor‐bearing C57BL/6 mice were treated consecutively for a total of four times, following the dosing regimen outlined in Figure 6A. To monitor the progress of the treatment, we measured and recorded the tumor volume and body weight of the mice every 2 days starting from the treatment day. The results showed that there was no significant change in the weight of mice treated with various treatments, further indicating the safety of APm/Ce6/HIF (Figure 6F). Tumor suppression of APm/Ce6/HIF+US was the most significant, with a tumor suppression rate of 89.8%, while the tumor suppression rates of APm/Ce6/HIF and APm/Ce6+US were 41.8% and 41.3% (Figure 6G,H). On the 15th day, tumors from mice were collected and weighed. It was found that the mean tumor weight of mice treated with APm/Ce6/HIF+US was 0.24 ± 0.04 g, which was significantly less than that treated with PBS (0.92 ± 0.24 g) and APm/Ce6+US (0.40 ± 0.10 g) (Figure 6I). This showed that there was a better antitumor effect of APm/Ce6/HIF+US. Subsequently, hematoxylin and eosin (H&E) staining was performed on the tumors of the mice, and from the results, it was shown that the therapeutic effect of the mice treated with APm/Ce6/HIF+US was the most effective among all treatments (Figure 6J). At the same time, immunofluorescence staining indicated that the synergistic therapeutic effect of APm/Ce6/HIF+US was achieved by inhibiting the expression of HIF‐2α (Figure 6K).

### 2.8 APm/Ce6/HIF+US Reprograms the TME to Immunity

DAMPs released during ICD promoted the maturation of DCs and the infiltration of CD8^+^ T cells, subsequently causing changes in the TME and enhancing the antitumor immunity.^[^ ^24^ ^]^ To further investigate the effect of APm/Ce6/HIF+US on the tumor immune microenvironment, we collected the tumor‐draining lymph nodes (TDLNs), spleens and tumors from the mice treated with various treatments, and the population of immune cells was analyzed with FCM.

**Figure 7:**
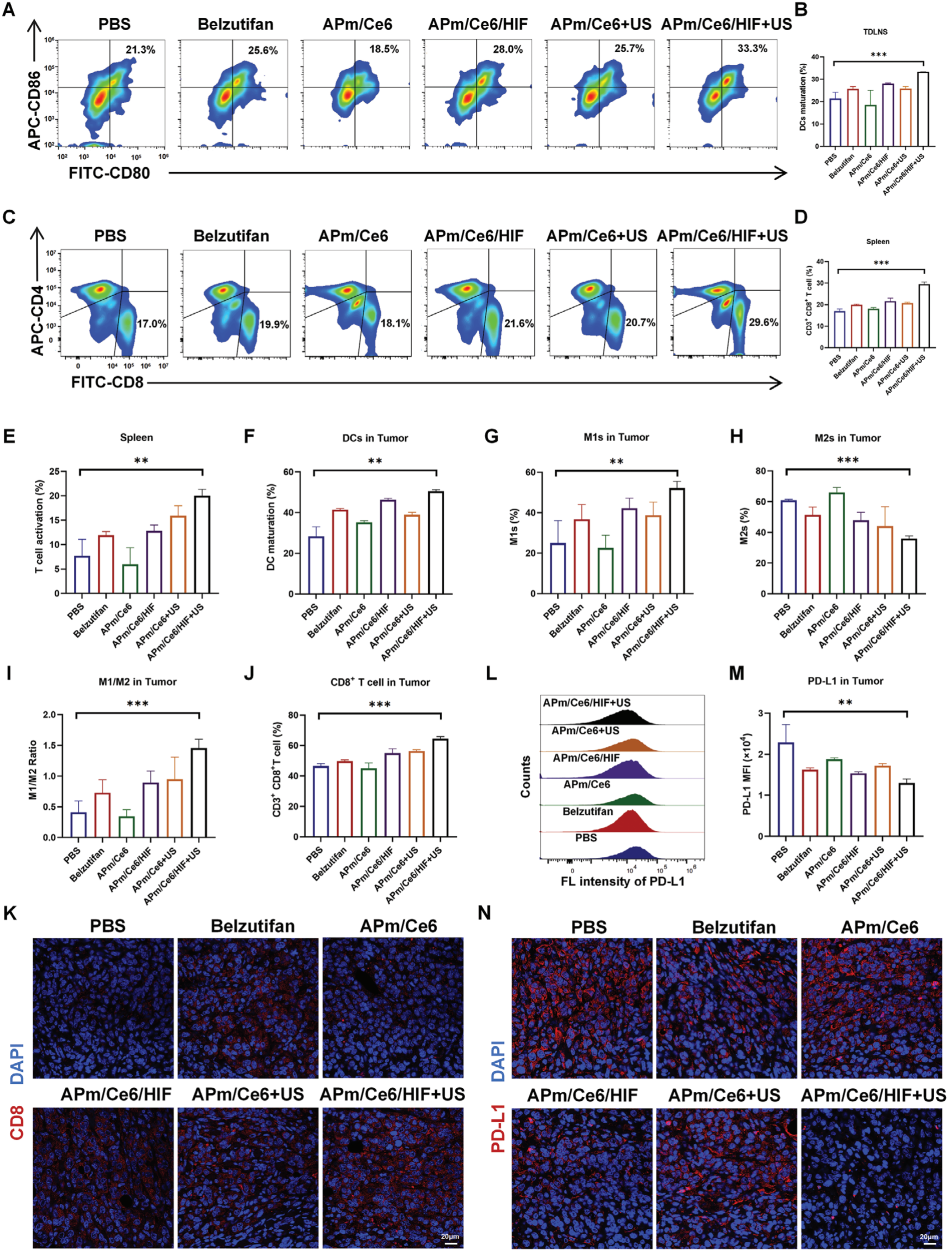
APm/Ce6/HIF+US reprograms the TME to activate antitumor immunity. A,B) The maturation of DCs (CD11C^+^ CD80^+^ CD86^+^) in TDLNs of mice treated with various treatments was detected by FCM (n = 5). C,D) The percentage of cytotoxic T lymphocytes (CD3^+^ CD8^+^) in the spleen of mice treated with various treatments was detected by FCM (n = 5). E) The percentage of T cell activation (CD3^+^ CD8^+^ CD69^+^) in the spleen of mice treated with various treatments was detected by FCM (n = 5). F) The percentage of mature DCs (CD11C^+^ CD80^+^ CD86^+^) in tumors of mice treated with various treatments were detected by FCM (n = 5). G–I) The percentage of M1 (F4/80^+^ CD80^+^) and M2 (F4/80^+^ CD206^+^) macrophages and their population in tumors of mice treated with various treatments were detected by FCM (n = 5). J) The percentage of cytotoxic T lymphocytes (CD3^+^ CD8^+^) in tumors of mice treated with various treatments were detected by FCM (n = 5). K) The expression of CD8 in T cells in tumor tissue of mice treated with various treatments was detected by CLSM. Scale bar: 20 µm. L,M) The expression of PD‐L1 (CD274^+^) in cancer cells of mice treated with various treatments was detected by FCM (n = 5). N) The expression of PD‐L1 in tumor tissue of mice treated with various treatments was detected by CLSM. Scale bar: 20 µm. Data are presented as mean ± SD. Statistical significance was calculated by one‐way analysis of variance. **p < 0.01, ***p < 0.001.

As shown in **Figure** **7**A,B, the proportion of mature DCs within TDLNs of mice treated with APm/Ce6/HIF+US (33.3%) was 1.56 times higher than that in mice treated with PBS (21.3%). Additionally, the population of CD3^+^ CD8^+^ T lymphocytes and the activation of T cells were 1.74 and 2.59 times higher in spleens of mice treated with APm/Ce6/HIF+US than those of mice treated with PBS (Figure 7C–E). The systemic immune responses activated by DCs maturation were also assessed by examining the tumor infiltration of DCs, macrophages, and T cells. FCM results showed that the relative proportion of mature DCs in tumor tissues of mice treated with APm/Ce6/HIF+US (50.6%) was higher than that of mice treated with PBS (28.3%) by 22.3% (Figure 7F). Tumor‐associated macrophages (TAMs) represent the most abundant innate immune cell population in the TME. The results showed that the proportions of M1‐type macrophages and M2‐type macrophages in the tumor tissues of mice treated with APm/Ce6/HIF+US were 52.1% and 35.9%, respectively, with a 108.5% increase in M1‐type macrophages and a 41.1% decrease in M2‐type macrophages compared to that in mice treated with PBS (Figure 7G–I). Mature DCs can further present and release antigens and activate T cells in the tumor, thus promoting antitumor immune responses. As expected, the proportion of CD3^+^ CD8^+^ T cells in the tumor tissues of mice treated with APm/Ce6/HIF+US was 64.5%, representing an 18.0% increase compared with that in mice treated with PBS (46.5%) (Figure 7J). In addition, immunofluorescence staining also revealed that the infiltration of effector T cells was higher in the tumor tissues of mice treated with APm/Ce6/HIF+US (Figure 7K). Considering that belzutifan significantly inhibited the expression of PD‐L1 in cancer cells in vitro (Figure 5G,H), the expression of PD‐L1 in the tumors was also examined. It was found that the expression of PD‐L1 was decreased in the tumor tissues of mice treated with APm/Ce6/HIF+US, which was 43.2% lower than that in the mice treated with PBS (Figure 7L,M). In addition, to visualize the expression of PD‐L1 in tumor tissues, immunofluorescence staining was performed, revealing that there was more down‐regulation of PD‐L1 expression (red fluorescence) in the tumor tissues of mice treated with APm/Ce6/HIF+US (Figure 7N). Taken together, we found that APm/Ce6/HIF+US can reprogram the TME to activate antitumor immune responses.

### 2.9 APm/Ce6/HIF+US Synergized with αPD‐1 to Enhance the Antitumor Efficacy

In recent years, the targeting of ICIs such as PD‐1, PD‐L1, and CTLA4 has emerged as a crucial approach in tumor treatment.^[^ ^25^ ^]^ αPD‐1 can competitively bind to PD‐1 on the surface of T cells with PD‐L1 of cancer cells, thereby enhancing the ability of T cells to kill cancer cells. It has been observed that the therapeutic efficacy of αPD‐1 is constrained by the presence of an immunosuppressive TME, which limits the effectiveness of αPD‐1 monotherapy in tumor treatment.^[^ ^26^ ^]^

**Figure 8:**
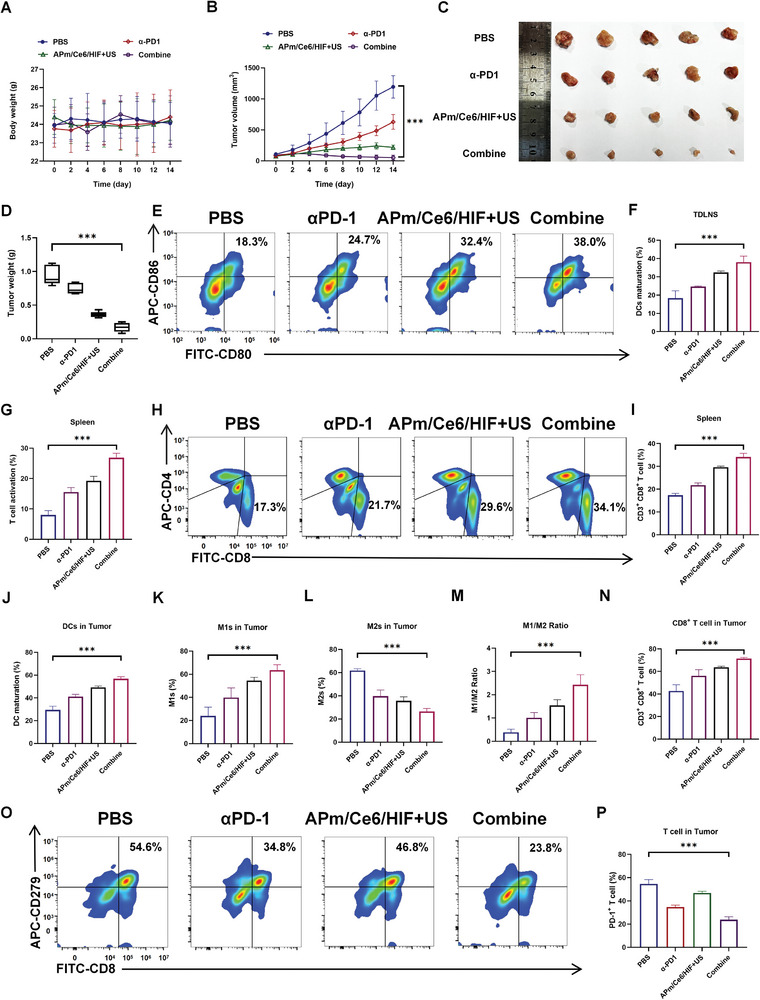
APm/Ce6/HIF+US combined with αPD‐1 synergistically enhances anticancer efficacy. A) The weight of mice treated with PBS, αPD‐1, APm/Ce6/HIF+US, and αPD‐1+APm/Ce6/HIF+US (n = 5). B–D) (B) Curve of tumor growth of mice treated with various treatments, (C) tumor images, and (D) tumor weight of mice treated with various treatments (n = 5). E,F) The percentage of DCs (CD11C^+^ CD80^+^ CD86^+^) in TDLNs of mice treated with various treatments was detected by FCM (n = 5). G) The percentage of T cell activation in the spleen of mice treated with various treatments was detected by FCM (n = 5). H,I) The percentage of cytotoxic T lymphocytes in the spleen of mice treated with various treatments was detected by FCM (n = 5). J) The percentage of mature DCs (CD11C^+^ CD80^+^ CD86^+^) in tumors of mice treated with various treatments were detected by FCM (n = 5). K–M) Histogram depicting the percentage of (K) M1 (F4/80^+^ CD80^+^) and (L) M2 (F4/80^+^ CD206^+^) macrophages in tumors after various treatments were detected by FCM and their (M) proportional relationship. N) Histogram depicting the percentage of cytotoxic T lymphocytes (CD3^+^ CD8^+^) in tumors after various treatments were detected by FCM (n = 5). O,P) The expression of PD‐1 (CD3^+^ CD8^+^ CD279^+^) on the surface of infiltrating T cells in tumors after various treatments was detected by FCM (n = 5). Data are presented as mean ± SD. Statistical significance was calculated by one‐way analysis of variance. ***p < 0.001.

The results above demonstrate that APm/Ce6/HIF+US not only reduced the expression of PD‐L1 but also reshaped the tumor immune microenvironment. Therefore, we hypothesized that the combination of APm/Ce6/HIF+US and αPD‐1 could synergistically enhance antitumor efficacy. To further investigate the antitumor efficacy of APm/Ce6/HIF+US combined with αPD‐1, RENCA tumor‐bearing mice were established. PBS, αPD‐1, APm/Ce6/HIF+US, and αPD‐1+APm/Ce6/HIF+US were used to treat RENCA tumor‐bearing mice. It was found that the weight of mice treated with various treatments did not change (**Figure** **8**A). Every other day after administration, the tumor volume and body weight of the mice were measured. The results showed that mice treated with PD‐1+APm/Ce6/HIF+US showed significant inhibition of tumor growth, compared with that in mice treated with PBS, αPD‐1, and APm/Ce6/HIF+US, respectively (Figure 8B,C). Specifically, the tumor volume of mice treated with PBS, αPD‐1, APm/Ce6/HIF+US, and αPD‐1+APm/Ce6/HIF+US was 1194.2, 628.5, 218.4, and 52.9 mm^3^, respectively, indicating that αPD‐1+APm/Ce6/HIF+US had the best antitumor effect. Then, the mice were sacrificed, and their tumors were collected for weighing. It was found that the average tumor weight of mice treated with αPD‐1+APm/Ce6/HIF+US was only 0.17 g, which were 0.95, 0.75, and 0.36 g for the mice treated with PBS, αPD‐1, and APm/Ce6/HIF+US, respectively (Figure 8D). Taken together, these results indicated that αPD‐1+APm/Ce6/HIF+US had the strongest tumor suppressive effect in vivo.

To further explore the effect of αPD‐1+APm/Ce6/HIF+US on the TME, the infiltration of immune cells in the TDLNs, spleens, and tumor tissues was then analyzed. It was found that the maturation of DCs in TDLNs of mice treated with αPD‐1+APm/Ce6/HIF+US was 38.0%, which was ≈2.1 times higher than that in the spleen of mice treated with PBS (18.3%) (Figure 8E,F). Second, the proportion of T cell activation in spleens of mice treated with αPD‐1+APm/Ce6/HIF+US was 26.9%, which was 3.4 times higher than that in mice treated with PBS (8.0%) (Figure 8G). And the proportion of CD3^+^ CD8^+^ T cells in the spleens of mice treated with αPD‐1+APm/Ce6/HIF+US was 34.1%, representing a 16.8% increase compared with that in mice treated with PBS (17.3%) (Figure 8H,I). Furthermore, the relative proportion of mature DCs in tumor tissues of mice treated with αPD‐1+APm/Ce6/HIF+US (56.8%) was higher than that of mice treated with PBS (29.4%) by 27.4% (Figure 8J). Mature DCs, as the most potent antigen‐presenting cells, interact with myriad immune cells, resulting in macrosphage polarization and differentiation of naive T cells into CD8^+^ T cells. FCM analysis showed that the proportion of M1‐type macrophages increased, corresponding to decreased M2‐type macrophage population in the tumors of mice treated with αPD‐1+APm/Ce6/HIF+US (M1‐type macrophages: αPD‐1+APm/Ce6/HIF+US vs PBS = 63.5% vs 24.0%; M2‐type macrophages: αPD‐1+APm/Ce6/HIF+US vs PBS = 26.5% vs 61.9; M1/M2 ratio: αPD‐1+APm/Ce6/HIF+US vs PBS = 2.43 vs 0.39) (Figure 8K–M). Furthermore, the population of cytotoxic T cells in the tumors of mice treated with various treatments was also examined. The proportion of CD3^+^ CD8^+^ T cells infiltrated in the tumor tissues of mice treated with αPD‐1+APm/Ce6/HIF+US was 71.3%, representing a 28.6% increase compared with that in mice treated with PBS (42.7%) (Figure 8N). To verify the synergistic therapeutic effect of αPD‐1, the expression level of PD‐1 on the surface of T cells was examined. It was found that the expression of PD‐1 on the surface of T cells was decreased significantly after αPD‐1+APm/Ce6/HIF+US treatment (αPD‐1+APm/Ce6/HIF+US vs PBS = 23.8% vs 54.6%) (Figure 8O,P), suggesting that αPD‐1 promoted the antitumor efficacy of APm/Ce6/HIF+US by reducing the expression of PD‐1 on the surface of T cells. In summary, APm/Ce6/HIF+US synergized with αPD‐1 to reprogram TME and trigger effective antitumor immunity.

## 3 Conclusion

In summary, we designed a hypoxia‐responsive polymer (P‐APm) loaded with belzutifan and Ce6, forming APm/Ce6/HIF through self‐assembly. APm/Ce6/HIF, when injected systemically, preferentially accumulate in tumor tissues. In the hypoxic TME, APm/Ce6/HIF rapidly degrade, releasing belzutifan and Ce6. The released belzutifan effectively suppresses tumor growth by targeting hypoxia and autophagy signaling pathways. Furthermore, the Ce6 enhances SDT, significantly increasing the generation of ROS and triggering ICD. This process reprograms the “immune‐cold” tumor into “immune‐hot” tumor by promoting DCs maturation and tumor infiltration of cytotoxic T cells and M1 macrophages, leading to a synergistic effect when combined with αPD‐1 therapy. Overall, our study presents a promising targeted therapy combined with immunotherapy strategy for the treatment of RCC, demonstrating significant clinical application potential.

## 4 Experimental Section

### Materials and Reagents

Belzutifan was purchased from Bide Pharm (Shanghai, Chian). 4‐nitrobenzyl alcohol, n, n‐dimethyl formamide, polyethylene glycol monomethyl ether (mPEG_5k_), 3‐(4,5‐dimethylthiazol‐2‐yl) ‐2,5‐ diphenyltetrazolium bromide (MTT), and sodium dodecyl sulfate (SDS) were purchased from Aladdin Co., Ltd. (Shanghai, China). 1,2,4,5‐cyclohexanetetracarboxylic dianhydride (PMDA) was purchased from Energy‐chemical (Shanghai, China). Cy5.5 and Cy7.5 were purchased from Solarbio, Co., Ltd. (Beijing, China). The primary antibodies used, including anti‐CD11c‐PE, anti‐CD80‐FITC, anti‐CD86‐APC, anti‐CD69‐APC, anti‐CD3‐PE, anti‐CD4‐APC, anti‐CD8‐FITC, anti‐F4/80‐PE, anti‐CD206‐APC, anti‐CD274‐PE and anti‐CD279‐APC were all purchased from BioLegend, Co., Ltd. (USA). Cell culture vessels were purchased from NEST (Wuxi, China). Dulbecco's modified Eagle's medium (DMEM), RPMI‐1640 medium, penicillin/streptomycin (P/S), 0.25% trypsin‐EDTA, and fetal bovine serum (FBS) were purchased from Gibco (Grand Island, NY, USA). Annexin V‐FITC/PI Cell Apoptosis Kit, Calcein/PI Live/Dead Viability/Cytotoxicity Assay Kit, and reactive oxygen species (ROS) assay kit were purchased from Beyotime Biotechnology (Shanghai, China).

### General Measurements

^1^H NMR spectra were measured by a 300 and 400 MHz NMR spectrometer (Bruker) at room temperature. The morphology and size of nanoparticles were obtained by transmission electron microscopy (TEM) carried out with an HT7700 electron microscope. Size and zeta potential measurements were conducted on a Malvern Zetasizer (Nano ZS, UK). The absorbance spectra and fluorescence spectra were measured using a UV–vis Spectrophotometer (UV‐2600) and fluorescence spectrometer (FLS980), respectively. Immunofluorescence images were performed using confocal laser scanning microscopy (CLSM, FV1000‐IX81, Olympus, Japan). The MTT and CCK8 assays were conducted using a Microplate reader (Spectra Max M3). The mice imaging was conducted using an in vivo Imaging System (IVIS, PerkinElmer). Flow cytometry (FCM) was analyzed using a flow cytometry analyzer (Beckman Coulter, USA).

### Cell Culture

786‐O, A498, and RENCA cells were cultured in 1640 medium supplemented with 10% fetal bovine serum, 100 µg mL^−1^ penicillin and 100 µg mL^−1^ streptomycin at 37 °C with 5% (v/v) CO_2_. The ACHN cells were cultured in DMEM medium supplemented with 10% fetal bovine serum, 100 µg mL^−1^ penicillin and 100 µg mL^−1^ streptomycin at 37 °C with 5% (v/v) CO_2_. When the cell density reached 70–80%, the cells were digested with 0.25% trypsin and then sub‐cultured or inoculated in cell plates for subsequent experiments.

### Synthesis of 4,4′‐Azobisbenzenemethanol

Under ice bath conditions, NaOH (11.4 g, 285 mmol) was dissolved in 50 mL of ultrapure water. The mixture was stirred until NaOH was completely dissolved. 4‐nitrobenzyl alcohol (2 g, 13.06 mmol) was added to the solution, and the solution turned yellow. Under stirring, Zn powder (3 g, 46.1 mmol) was then added to the solution, and the solution turned from yellow to green. The solution was then cooled to room temperature, and the color changed back to yellow. The solution was then heated to 85 °C and kept for 12 h under reflux. After the reaction, the mixture was filtered and the residue was collected. The collected solid was then dissolved in 100 mL of methanol and refluxed at 80 °C for 24 h. The reaction mixture was filtered, and the filtrate was collected and dried in the vacuum to obtain the product 4,4′‐azobisbenzenemethanol.

### Synthesis of P‐APm

4,4′‐azobisbenzenemethanol (605 mg, 2.5 mmol) and PMDA (615 mg, 2.75 mmol) were combined in a single‐necked flask. 20 mL of n, n‐dimethyl formamide was added to dissolve the mixture, which was then stirred at room temperature for 48 h. After this period, mPEG_5k_ (2.5 g, 0.5 mmol) was added and heated until dissolved. The resulting solution was stirred at room temperature for an additional 24 h. Finally, the polymer (Poly(4,4′‐azobisbenzenemethanol‐PMDA)‐mPEG_5k_, P‐APm) was obtained through dialysis and drying in the vacuum.

### Preparation and Characterization of APm/Ce6 and APm/Ce6/HIF

Belzutifan (10 mg), Chlorin e6 (Ce6, 6 mg), and P‐APm (100 mg) were first co‐dissolved in 1 mL dimethyl sulfoxide (DMSO). The supernatant was collected by centrifugal separation. The solution was dispersed in 10 mL of de‐ionized water with continuous stirring. After vigorous stirring for 15 min, the mixture was collected and dialyzed overnight using a dialysis bag with a molecular weight cut‐off of 3500 Da. The obtained APm/Ce6/HIF was stored at 4 °C for subsequent use. APm/Ce6 was synthesized using the same method as Ce6 (6 mg) and P‐APm (100 mg).

### RNA‐Sequencing Expression and Tumor Immune Dysfunction and Exclusion (TIDE) Algorithm

RNA‐sequencing expression (level 3) profiles and corresponding clinical information for RCC patients were downloaded from the TCGA dataset(https://portal.gdc.com). CD274, CTLA4, HAVCR2, LAG3, PDCD1, PDCD1LG2, TIGIT, and SIGLEC15 were selected to be immune‐checkpoint–relevant transcripts and the expression values of these eight genes were extracted. All the above analysis methods and R package were implemented by R Foundation for Statistical Computing (2020) version 4.0.3. Using the ggplot2 R package and pheatmap R package. Potential ICB response was predicted with TIDE algorithm.^[^ ^27^ ^]^

There are two different immune escape mechanisms in tumors: on the one hand, some immunosuppressive factors can prevent the invasion of T cells; on the other hand, some tumors have high levels of cytotoxic T cell invasion, but these T cells are in a functional inactivation state. TIDE algorithm predicts tumor immune escape by assessing the activity of both mechanisms together. Higher TIDE scores were associated with poorer immune checkpoint suppression therapy. TIDE is a computational tool used to assess tumor immune escape mechanisms and predict immune checkpoint inhibitor (ICI) response. TIDE calculations are done directly using the TIDE scoring online tool (http://tide.dfci.harvard.edu/).

### Cytotoxicity Evaluation

The cytotoxicity of APm/Ce6/HIF was detected by MTT assay. In brief, 786‐O cells were seeded in 96‐well plates (5 × 10^3^ cells/well) overnight and then treated with different treatments, including PBS, belzutifan, APm/Ce6, APm/Ce6/HIF, APm/Ce6+US and APm/Ce6/HIF+US. The concentrations of belzutifan and Ce6 were consistent, varying from 3.125 to 100 µM for belzutifan and 1.875 to 60 µg mL^−1^ for Ce6, respectively. After a 6‐h treatment period, the cells treated with APm/Ce6+US or APm/Ce6/HIF+US were exposed to ultrasound (1 W cm^−2^, frequency of 1 MHz, duty ratio of 50%) for 2 min. Following 12 h of incubation, a fresh medium containing MTT was added, and the cells were further incubated for 4 h. Then 200 µL of SDS per well was added. The optical density (OD) value at a wavelength of 570 nm was measured using a microplate system.

### CCK8 Assay

786‐O, A498, and ACHN cells were seeded overnight in 96‐well plates (5 × 10^3^ cells/well) and then treated with DMSO and belzutifan (the concentrations of belzutifan were 50 µm). Afterward, the cells treated in the normoxic environment were cultured at 37 °C with 5% (v/v) CO_2_ and 21% (v/v) O_2_, while the cells treated in the hypoxic environment were cultured at 37 °C with 5% (v/v) CO_2_ and 1% (v/v) O_2_. After 24, 48, and 72 h of incubation, fresh medium containing CCK8 was added and incubation was continued for 2 h. The OD values at a concentration of 450 nm were measured using a microplate system.

### Intracellular ROS Generation

The generation of intracellular ROS was assessed using H_2_DCFDA. Initially, 786‐O cells were plated evenly and then treated with PBS, belzutifan, APm/Ce6, APm/Ce6/HIF, APm/Ce6+US and APm/Ce6/HIF+US (the concentrations of Ce6 and belzutifan were 30 µg mL^−1^ and 50 µm, respectively) for 6 h and then incubated with H_2_DCFDA for 30 min. Afterward, the cells treated with APm/Ce6+US or APm/Ce6/HIF+US were exposed to ultrasound (1 W cm^−2^, frequency of 1 MHz, duty ratio of 50%) for 2 min. After being washed twice with PBS, the cells were stained with DAPI for 10 min. The fluorescence images were examined by CLSM, and the production of ROS was quantified by FCM.

### Cell Apoptosis Assay

786‐O cells were cultured in 6‐well plates (1 × 10^6^ cells/well) overnight and then treated with PBS, belzutifan, APm/Ce6, APm/Ce6/HIF, APm/Ce6+US and APm/Ce6/HIF+US (the concentrations of Ce6 and belzutifan were 30 µg mL^−1^ and 50 µm, respectively) for 6 h. Afterward, the cells treated with APm/Ce6+US or APm/Ce6/HIF+US were exposed to ultrasound (1 W cm^−2^, frequency of 1 MHz, duty ratio of 50%) for 2 min. After a 12‐h incubation, the cells were collected and re‐suspended in PBS containing Annexin V‐FITC and propidium iodide (PI) for 15 min in a dark environment. Finally, apoptosis was detected by FCM.

### Live/Dead State Detection

Live/dead state detection was performed using Calcein‐AM/PI staining kits. 786‐O cells were cultured in confocal dishes (2 × 10^5^ cells/well) overnight and then treated with PBS, belzutifan, APm/Ce6, APm/Ce6/HIF, APm/Ce6+US and APm/Ce6/HIF+US (the concentrations of Ce6 and belzutifan were 30 µg mL^−1^ and 50 µm, respectively) for 6 h. Afterward, the cells treated with APm/Ce6+US or APm/Ce6/HIF+US were exposed to ultrasound (1 W cm^−2^, frequency of 1 MHz, duty ratio of 50%) for 2 min. After incubation for 12 h, the cells were cultured with Calcein‐AM/PI in buffer solutions for 15 min. Subsequently, images were collected with CLSM.

### Colony Formation Assays

786‐O cells were seeded in 6‐well plates (1 × 10^3^ cells/well) and cultured for 24 h. Subsequently, the cells were treated with PBS, belzutifan, APm/Ce6, APm/Ce6/HIF, APm/Ce6+US and APm/Ce6/HIF+US (the concentrations of Ce6 and belzutifan were 30 µg mL^−1^ and 50 µm, respectively) for 6 h. Afterward, the cells treated with APm/Ce6+US or APm/Ce6/HIF+US were exposed to ultrasound (1 W cm^−2^, frequency of 1 MHz, duty ratio of 50%) for 2 min. Then the medium was refreshed and incubated for 7 days before being stained with 0.1% crystal violet.

### Observation of Autophagosome and Autophagolysosome

786‐O cells were seeded in 6‐well plates (1 × 10^6^ cells/well) and cultured for 24 h. Subsequently, the cells were treated with PBS, belzutifan, APm/Ce6, APm/Ce6/HIF, APm/Ce6+US and APm/Ce6/HIF+US (the concentrations of Ce6 and belzutifan were 30 µg mL^−1^ and 50 µM, respectively) for 6 h. Afterward, the cells treated with APm/Ce6+US or APm/Ce6/HIF+US were exposed to ultrasound (1 W cm^−2^, frequency of 1 MHz, duty ratio of 50%) for 2 min. After incubation for 12 h, the cells were collected, fixed by electron microscope fixative (G1102, Servicebio), and observed by TEM.

### Measurement of Cell Surface Calreticulin (CRT)

For CLSM analysis of CRT exposure, 786‐O cells were seeded on a live cell imaging glass bottom dish (1 × 10^5^ cells/well). Subsequently, the cells were treated with PBS, belzutifan, APm/Ce6, APm/Ce6/HIF, APm/Ce6+US and APm/Ce6/HIF+US (the concentrations of Ce6 and belzutifan were 30 µg mL^−1^ and 50 µm, respectively) for 6 h. Afterward, the cells treated with APm/Ce6+US or APm/Ce6/HIF+US were exposed to ultrasound (1 W cm^−2^, frequency of 1 MHz, duty ratio of 50%) for 2 min. Followed by incubation for 6 h, cells were washed with PBS and fixed with 100% methanol at room temperature for 5 min. Then, cells were rewashed with PBS and permeabilized with 0.1% Triton X‐100 for 5 min. After blocked with 1% BSA for 1 h, cells were incubated with CRT primary antibody (ab211962, Abcam) diluted in blocking buffer overnight. The following day, cells were washed thrice with PBS and incubated with Alexa fluor 488 secondary antibody for 45 min. Finally, the cell membrane was labeled in red by Actin‐Tracker Red‐555 (C2203S, Beyotime), nuclear DNA was labeled in blue with DAPI, and images were taken by CLSM.

FCM was used for the surface detection of CRT. 786‐O cells were seeded in the 12‐well plate (3 × 10^5^ cells/well). Subsequently, the cells were treated as described above. Afterward, the cells were collected and blocked with 1% BSA and then incubated with CRT primary antibody. After washing with PBS 3 times, cells were incubated with Alexa Fluor 488‐conjugated antibody for 30 min and then analyzed by FCM.

### Measurement of the Release of High Mobility Group Box 1 (HMGB1)

For CLSM analysis of HMGB1 release, 786‐O cells were seeded on a live cell imaging glass bottom dish (1 × 10^5^ cells/well). Subsequently, the cells were treated with PBS, belzutifan, APm/Ce6, APm/Ce6/HIF, APm/Ce6+US and APm/Ce6/HIF+US (the concentrations of Ce6 and belzutifan were 30 µg mL^−1^ and 50 µm, respectively) for 6 h. Afterward, the cells treated with APm/Ce6+US or APm/Ce6/HIF+US were exposed to ultrasound (1 W cm^−2^, frequency of 1 MHz, duty ratio of 50%) for 2 min. Followed by incubation for 12 h, cells were washed with PBS and fixed with 100% methanol at room temperature for 5 min. Then, cells were rewashed with PBS and permeabilized with 0.1% Triton X‐100 for 5 min. After blocked with 1% BSA for 1 h, cells were incubated with HMGB1 primary antibody (ab216986, Abcam) diluted in blocking buffer overnight. The following day, cells were washed thrice with PBS and incubated with Alexa fluor 555 secondary antibody for 45 min. Finally, the cell membrane was labeled green by Actin‐Tracker Green‐488 (C2201S, Beyotime), nuclear DNA was labeled blue with DAPI, and images were taken by CLSM.

### Bone‐Marrow‐Derived Dendritic Cells (BMDCs) Mature in Vitro

BMDCs were generated from male C57BL/6 mice and cultured in RPMI 1640 medium supplement with 10% FBS, granulocyte‐macrophage colony‐stimulating factor (GM‐CSF) (20 ng mL^−1^, Beyotime), and interleukin‐4 (IL‐4) (10 ng mL^−1^, Beyotime) at 37 °C with 5% (v/v) CO_2_. After 5 days of culturing, pretreated 786‐O cells were co‐incubated with BMDCs for 24 h. After treatment, DCs were stained with anti‐CD11c, anti‐CD80, and anti‐CD86 for DCs mature analysis via FCM.

### Measurement of Programmed Cell Death Protein Ligand‐1 (PD‐L1) Expression

To measure the expression of PD‐L1 in tumor cells, 786‐O cells were seeded on 12‐well plates (3 × 10^5^ cells/well). Subsequently, the cells were treated with PBS, belzutifan, APm/Ce6, APm/Ce6/HIF, APm/Ce6+US and APm/Ce6/HIF+US (the concentrations of Ce6 and belzutifan were 30 µg mL^−1^ and 50 µm, respectively) for 6 h. Afterward, the cells treated with APm/Ce6+US or APm/Ce6/HIF+US were exposed to ultrasound (1 W cm^−2^, frequency of 1 MHz, duty ratio of 50%) for 2 min. Followed by incubation for 12 h, the cells were stained by anti‐CD274 for 1 h in the dark and then washed with PBS three times. The fluorescence was immediately analyzed by FCM.

### Western Blot

Cell lysate (RIPA: protease inhibitor: phosphorylated protease inhibitor = 100: 2: 1) was added to the cell dish to extract all the proteins in the cells. The proteins were extracted by centrifugation at 12 000 rpm for 15 min. The concentration of the proteins was determined using a BCA protein assay kit (P0011, Beyotime). Forty micrograms of protein sample were added per lane, and the samples were separated on a 10% sodium dodecyl sulfate‐polyacrylamide gel electrophoresis (SDS‐PAGE) and transferred to the PVDF membrane by a gel‐electrophoretic apparatus (Bio‐Rad mini, USA). After blocking for one h with 5% milk or 5% BSA, membranes were incubated with primary antibody overnight at 4 °C. Subsequently, the PVDF films were washed 3 times and incubated with secondary antibodies for 2 h at room temperature. The Western blot images were obtained by Chemiluminescence imaging system (Servicebio, SCG‐W2000, China) with 200 µL of ECL chemiluminescent reagent (KF001, Affinity) added on the top of the membrane. Antibodies used were: HIF‐2α (ab207607, Abcam), SQSTM1/p62 (39749, Cell Signaling Technology), LC3B (AL221, Beyotime), GAPDH (5174, Cell Signaling Technology).

### Animal Studies

All animal experiments reported herein were performed under the guidelines evaluated and approved by the Committee of Animal Experimentation and the Ethics Committee of Cancer Hospital, Chinese Academy of Medical Sciences (Approval number: NCC2024A013). C57BL/6 mice (male, 4 weeks old) were purchased from SPF Biotechnology (Beijing, China) and raised in SPF animal rooms.

### In Vivo Biodistribution Imaging

Once the RENCA tumor volumes reached 100 mm^3^, the mice were intravenously injected with Cy7.5‐labeled APm/Ce6/HIF (APm/Cy7.5), followed by imaging with IVIS (Spectrum CT, PerkinElmer, Ex/Em = 745 nm/840 nm) at 1, 4, 7, 12, 24, and 48 h post‐injection, respectively. At 48 h post‐injection, the tumor tissues and major organs (heart, liver, spleen, lung, kidney, intestine, and tumor) were collected and quantified using the IVIS.

### Establishment of RENCA Solid Tumor Model I and Therapeutic Effect

When the tumor volumes reached 100 mm^3^, RENCA tumor‐bearing mice were randomly divided into six groups followed by treated with PBS, belzutifan, APm/Ce6, APm/Ce6/HIF, APm/Ce6+US and APm/Ce6/HIF+US (APm/Ce6: 3 mg kg^−1^ Ce6 on days 0, 4, 8, 12, respectively; APm/Ce6/HIF: 3 mg kg^−1^ Ce6, 5 mg kg^−1^ belzutifan on days 0, 4, 8, and 12, respectively; ultrasound: 2 W cm^−2^, frequency of 1 MHz, duty ratio of 50% for 3min on days 1, 5, 9, and 13, respectively). The tumor volume and mouse weight were measured every other day, and the tumor volume (mm^3^) was calculated as V = (a × b^2^)/2, where a and b are the length and width of the tumor, respectively.

### Establishment of RENCA Solid Tumor Model II and Therapeutic Effect

When the tumor volumes reached 100 mm^3^, RENCA tumor‐bearing mice were randomly divided into 4 groups followed by treated with PBS, anti‐programmed cell death protein‐1 antibody (αPD‐1), APm/Ce6/HIF+US, and αPD‐1+APm/Ce6/HIF+US (APm/Ce6/HIF: 3 mg kg^−1^ Ce6, 5 mg kg^−1^ belzutifan on days 0, 4, 8, and 12, respectively; αPD‐1: 10 mg kg^−1^ on days 1, 5, 9, and 13, respectively; ultrasound: 2 W cm^−2^, frequency of 1 MHz, duty ratio of 50% for 3 min on days 1, 5, 9, and 13, respectively). The tumor volume and mouse weight were measured every other day, and the tumor volume (mm^3^) was calculated as V = (a × b^2^)/2, where a and b are the length and width of the tumor, respectively.

### Hematoxylin and Eosin (H&E) Staining and Immunofluorescence Analyses

The tissues were fixed in 4% paraformaldehyde neutral buffer and embedded in paraffin. Then the paraffin‐embedded tissues were cut into slices. According to the manufacturer's instructions, the slices were processed for immunohistochemical examination of H&E staining. Freshly dissected tumor tissues were fixed with 4% paraformaldehyde, dehydrated, embedded in paraffin wax, and transferred to the slice. Next, according to the manufacturer's protocol, the slices were incubated overnight with primary antibodies, HIF‐2α, CD8, and PD‐L1. After incubation with fluorescent secondary antibody and DAPI, the immunofluorescence images were captured using CLSM.

### Flow Cytometric Analysis

Fresh tumors, spleen, and draining lymph node tissue were collected for antitumor immune response analysis via FCM. Briefly, samples were dissociated into single‐cell suspensions. For characterizing T cells in the tumor and spleen, cells were stained by anti‐CD3, anti‐CD4, and anti‐CD8. To analyze DCs in the tumor and lymph nodes, cells were stained with anti‐CD11c, anti‐CD80, and anti‐CD86. To analyze tumor‐associated macrophages (TAMs) in the tumor, cells were stained for surface markers (anti‐F4/80 and anti‐CD80), then fixed and permeabilized using True‐Nuclear Transcription Factor Buffer Set (Bio Legend) and subsequently stained with anti‐CD206 antibody. To analyze activated T cells in the spleen, cells were stained for surface markers (anti‐CD3, anti‐CD8, and anti‐CD69). To analyze the expression of PD‐L1 in tumors, cells were stained with anti‐CD274. To analyze the expression of PD‐1 on T cells in tumors, cells were stained for surface markers (anti‐CD3, anti‐CD8, and anti‐CD279). All antibodies used above were purchased from BioLegend. CytExpert software was used for flow cytometer data acquisition and Flow Jo software was used for data processing.

### Statistical Analyses

All experiments were conducted three times or more independently (n ≥ 3). GraphPad Prism 9 software (GraphPad Software, Inc.) was used for statistical data analysis. To compare the differences between the two groups, Independent‐Samples t‐test was adopted. To compare the differences between multiple‐group means, a one‐way analysis of variance was used. P value less than 0.05 was considered statistically significant. Results were presented as means ± SD.

## Conflict of Interest

The authors declare no conflict of interest.

## Supporting information



Supporting Information

## Data Availability

The data that support the findings of this study are available from the corresponding author upon reasonable request.
