# Supplementary material for: Targeting Hypoxia and Autophagy Inhibition via Delivering Sonodynamic Nanoparticles With HIF‐2α Inhibitor for Enhancing Immunotherapy in Renal Cell Carcinoma
Source: Adv Healthc Mater. 2024 Oct 13;13(32):2402973. doi: 10.1002/adhm.202402973 (PMC11670269; doi:10.1002/adhm.202402973)
Supplement: Supplementary file 1 — Supporting Information [file ADHM-13-0-s001.docx]

Supporting Information

Targeting Hypoxia and Autophagy Inhibition *via* Delivering Sonodynamic Nanoparticles with HIF-2α Inhibitor for Enhancing Immunotherapy in Renal Cell Carcinoma

*Yihao Zhu^1#^, Yajian Li^1#^, Xuwen Li^1^, Yuan Yu^2^, Lingpu Zhang^3,4^, Hanchen Zhang^3,4^, Can Chen^5^, Dong Chen^1^, Mingshuai Wang^1^, Nianzeng Xing^1^, Feiya Yang^1*^, Wahafu Wasilijiang^1,6*^, Xiongjun Ye^1*^*

^1^ Department of Urology, National Cancer Center/National Clinical Research Center for Cancer/Cancer Hospital, Chinese Academy of Medical Sciences and Peking Union Medical College, Beijing 100021, China.

^2^ Zhejiang Cancer Hospital, Hangzhou Institute of Medicine, Chinese Academy of Sciences, Zhejiang 310022, China.

^3^ Beijing National Laboratory for Molecular Sciences, Laboratory of Polymer Physics and Chemistry, Institute of Chemistry, Chinese Academy of Sciences, Beijing 100190, China.

^4^ University of Chinese Academy of Sciences, Beijing 100049, China.

^5^ Department of Oncology, The Second Affiliated Hospital of Zunyi Medical University, Guizhou 563000, China.

^6^ Department of Urology, Shanxi Province Cancer Hospital/Shanxi Hospital Affiliated to Cancer Hospital Chinese Academy of Medical Sciences/Cancer Hospital Affiliated to Shanxi Medical University, Shanxi 030013, China.

**^#^ These authors contributed equally to this work.**

**Correspondence*:**

Xiongjun Ye, M.D. Department of Urology, National Cancer Center/National Clinical Research Center for Cancer/Cancer Hospital, Chinese Academy of Medical Sciences and Peking Union Medical College, No. 17, Panjiayuan Nanli, Chaoyang, 100021, Beijing, PR. China. Tel: +8613910380916. E-mail address: yexiongjun@cicams.ac.cn.

Wahafu Wasilijiang, M.D. Department of Urology, National Cancer Center/National Clinical Research Center for Cancer/Cancer Hospital, Chinese Academy of Medical Sciences and Peking Union Medical College, No. 17, Panjiayuan Nanli, Chaoyang, 100021, Beijing, PR. China. Tel: +8613810684425. E-mail address: wallonce@126.com.

Feiya Yang, M.D. Department of Urology, National Cancer Center/National Clinical Research Center for Cancer/Cancer Hospital, Chinese Academy of Medical Sciences and Peking Union Medical College, No. 17, Panjiayuan Nanli, Chaoyang, 100021, Beijing, PR. China. Tel: +8613691215799. E-mail address: [yangfeiya@cicams.ac.cn.](mailto:yangfeiya@cicams.ac.cn.%20)


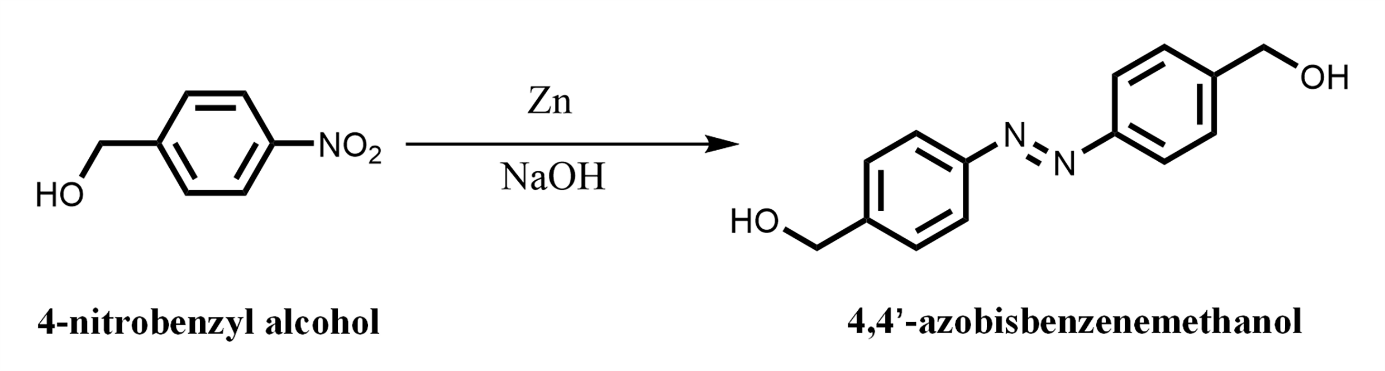
**Scheme S1: Synthesis of 4,4’-azobisbenzenemethanol.**

**
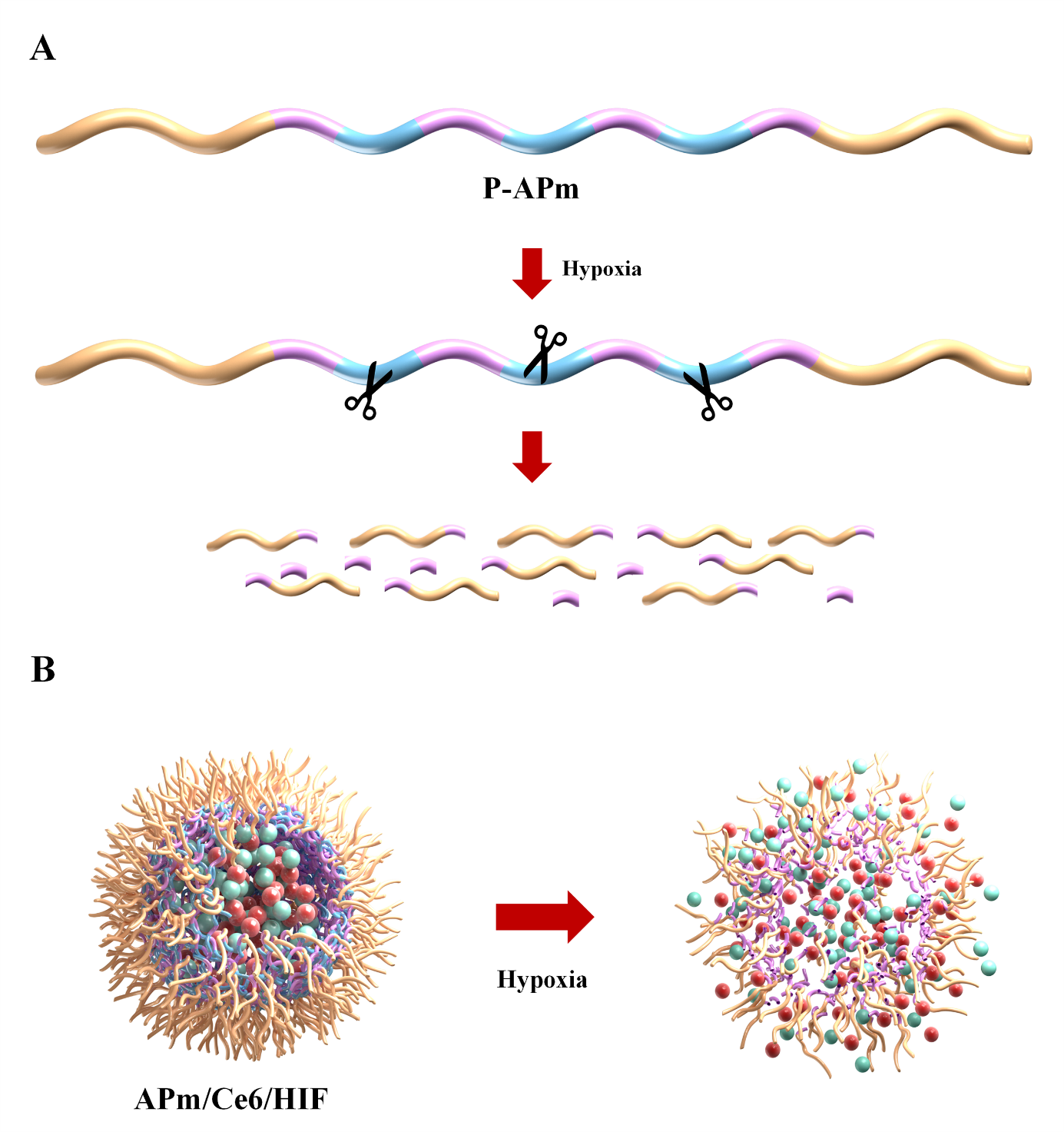
**

**Figure S1: Tumor hypoxia-specific degradation and drug release.** A: Degradation of P-APm in hypoxia tumor microenvironment (TME). B: Degradation of APm/Ce6/HIF in hypoxia TME.


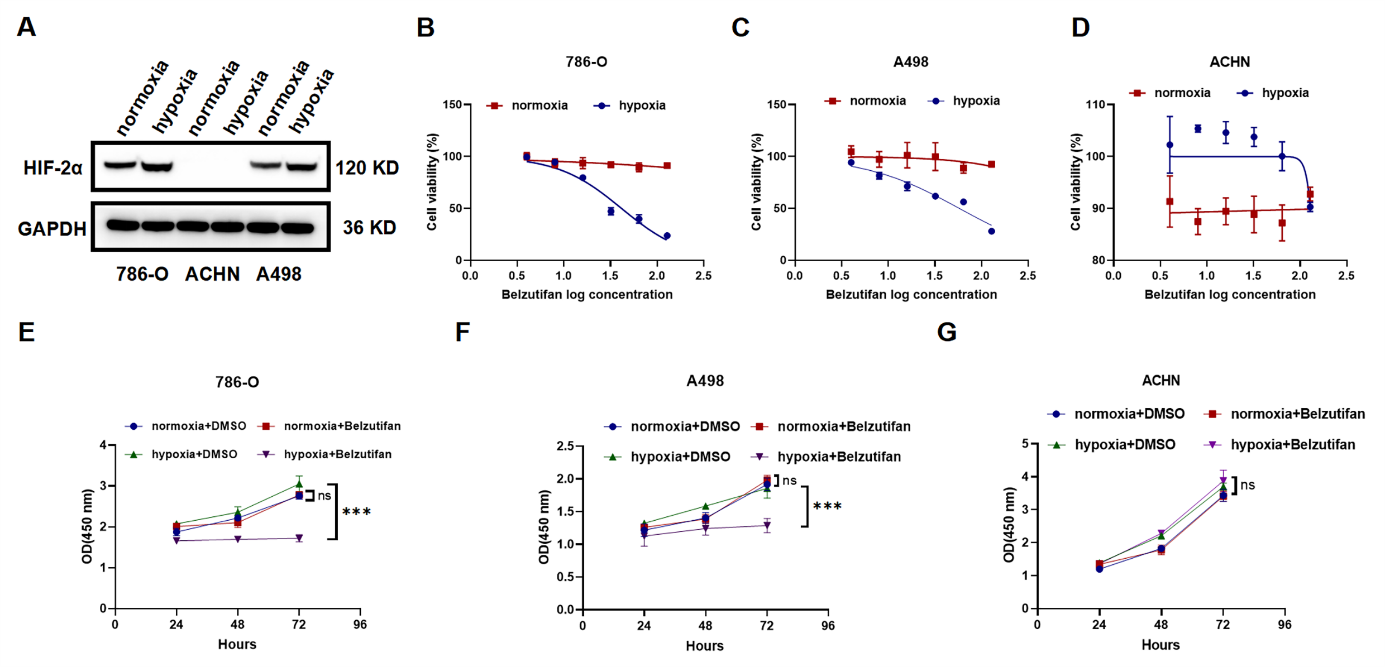
**Figure S2. Belzutifan significantly inhibited the proliferation of HIF-2α-expressing renal cell carcinoma (RCC) cells in the hypoxic environment.** A): Western blotting was performed to detect the expression of HIF-2α in RCC cells. B-D): MTT assay was performed to verify the effect of belzutifan on the viability of RCC cells (n=3). E-G): CCK8 assay was performed to verify the effect of belzutifan on the proliferation of RCC cells (n=3). Data are presented as mean ± SD. Statistical significance was calculated by one-way analysis of variance. ns, not significant, ****p* < 0.001.

**
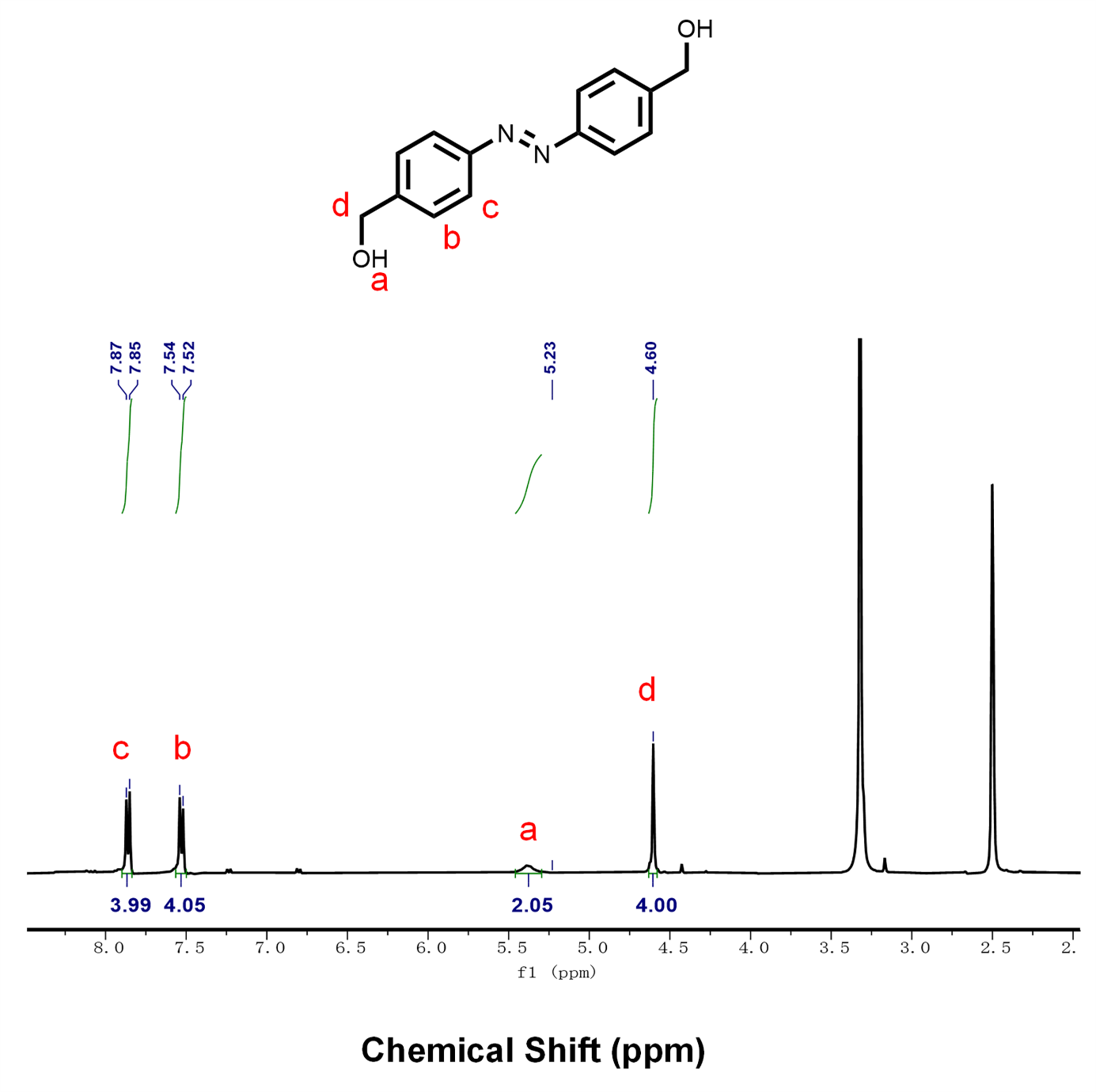
Figure S3. Characterization of 4,4'-azobisbenzenemethanol by ^1^H NMR.**


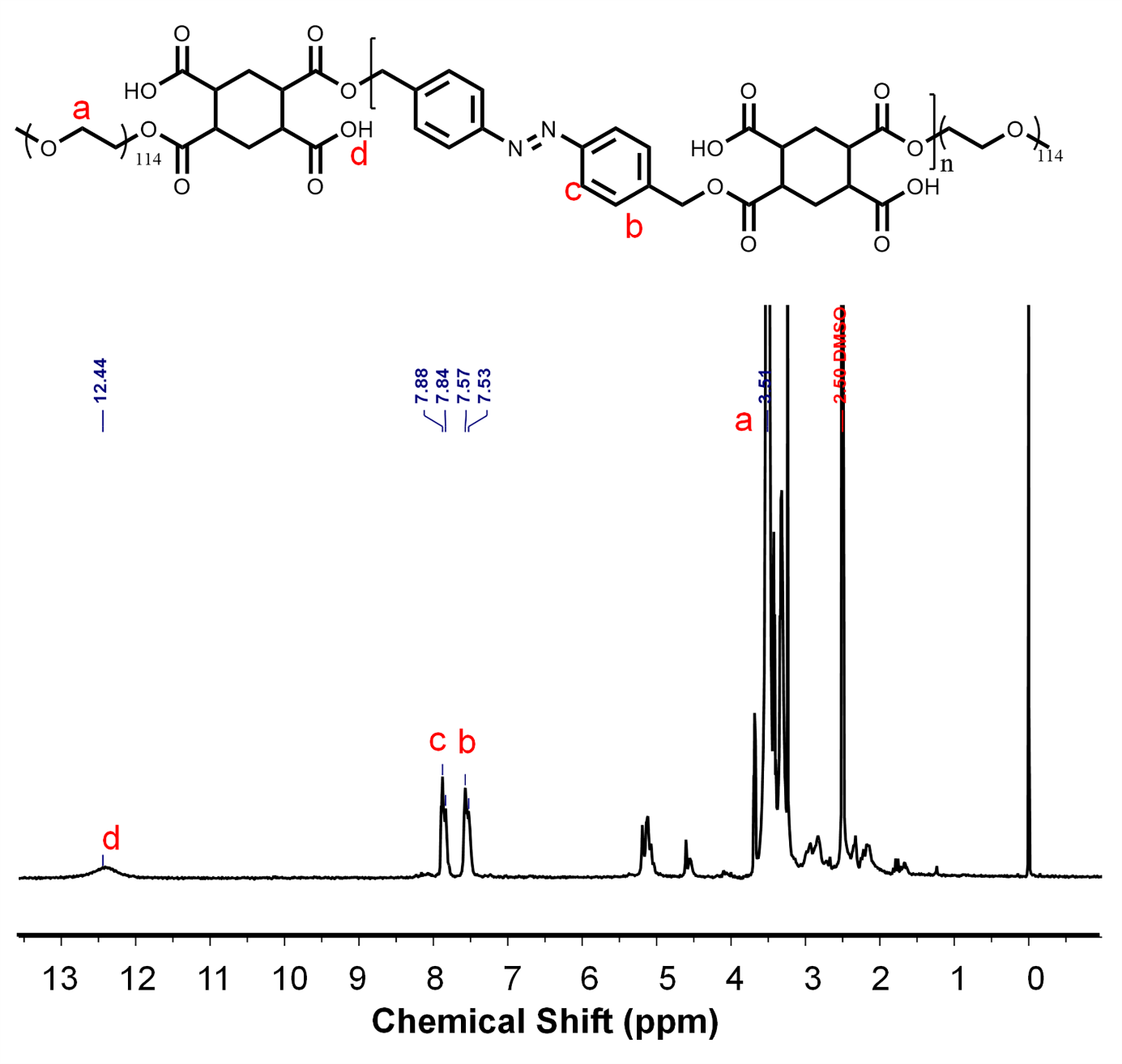
**Figure S4. Characterization of P-APm by ^1^H NMR.**

**
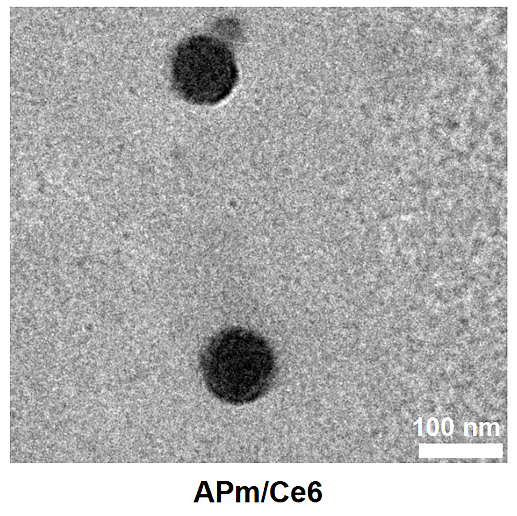
Figure S5. Characterization of APm/Ce6.** TEM was performed to detect the characterization of APm/Ce6. Scale bar: 100 nm.


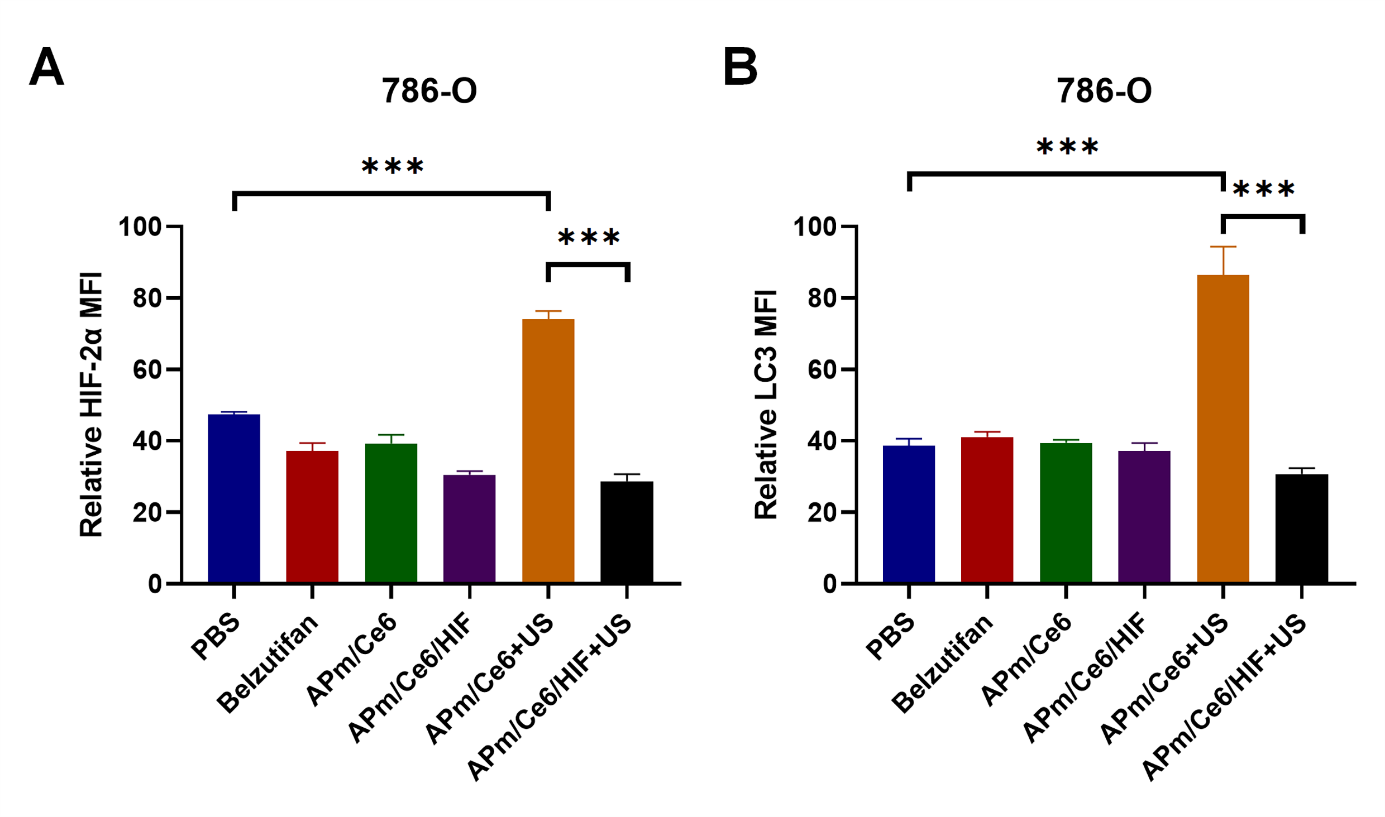
**Figure S6. APm/Ce6/HIF+US inhibited the proliferation of renal cancer cells by inhibiting hypoxia and autophagy signaling pathways.** A): The HIF-2α fluorescence intensity of 786-O cells was detected by ImageJ (n=3). B): The LC3 fluorescence intensity of 786-O cells was detected by ImageJ (n=3). Data are presented as mean ± SD. Statistical significance was calculated by one-way analysis of variance. ****p* < 0.001.

**Figure S7: The scheme of the autophagy signaling pathway in this study.** When RCC cells are treated with APm/Ce6+US, although it produces ICD to kill cancer cells, at the same time, the hypoxic TME activates autophagy to resist external killing effects on cells. But when RCC cells are treated with APm/Ce6/HIF+US, the released belzutifan further promotes cancer cell apoptosis by inhibiting the expression of HIF-2α and suppressing autophagy. Left bottom: autophagy signaling pathway. Autophagy refers to the formation of autophagosomes by the shedding of a bilayer membrane from the ribosome-free attachment area of the rough endoplasmic reticulum, which wraps around a portion of the cytoplasm and organelles, proteins, and other components that need to be degraded within the cell. Further autophagosomes fuse with lysosomes to form autophagolysosomes, which degrade the contents they contain.
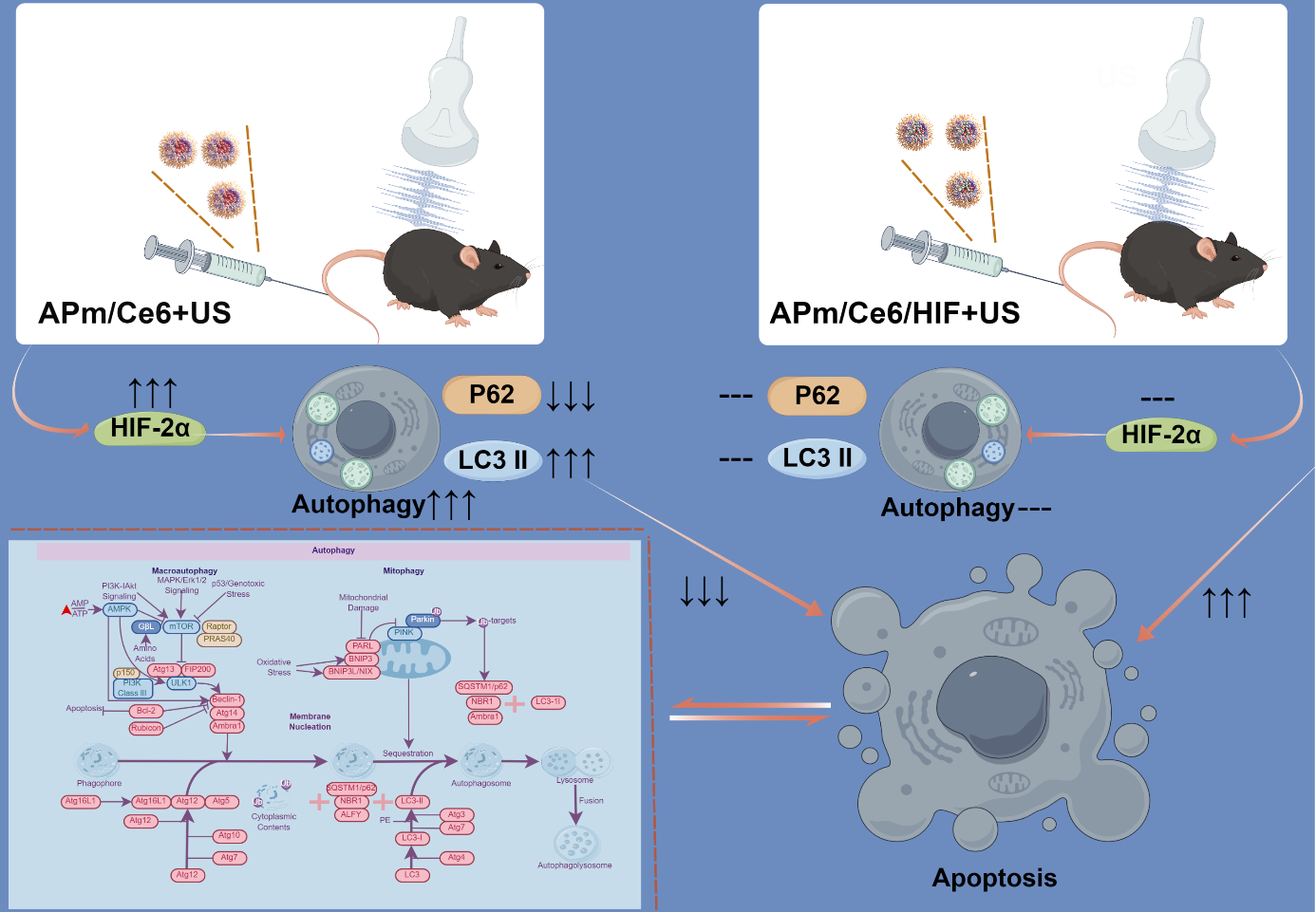


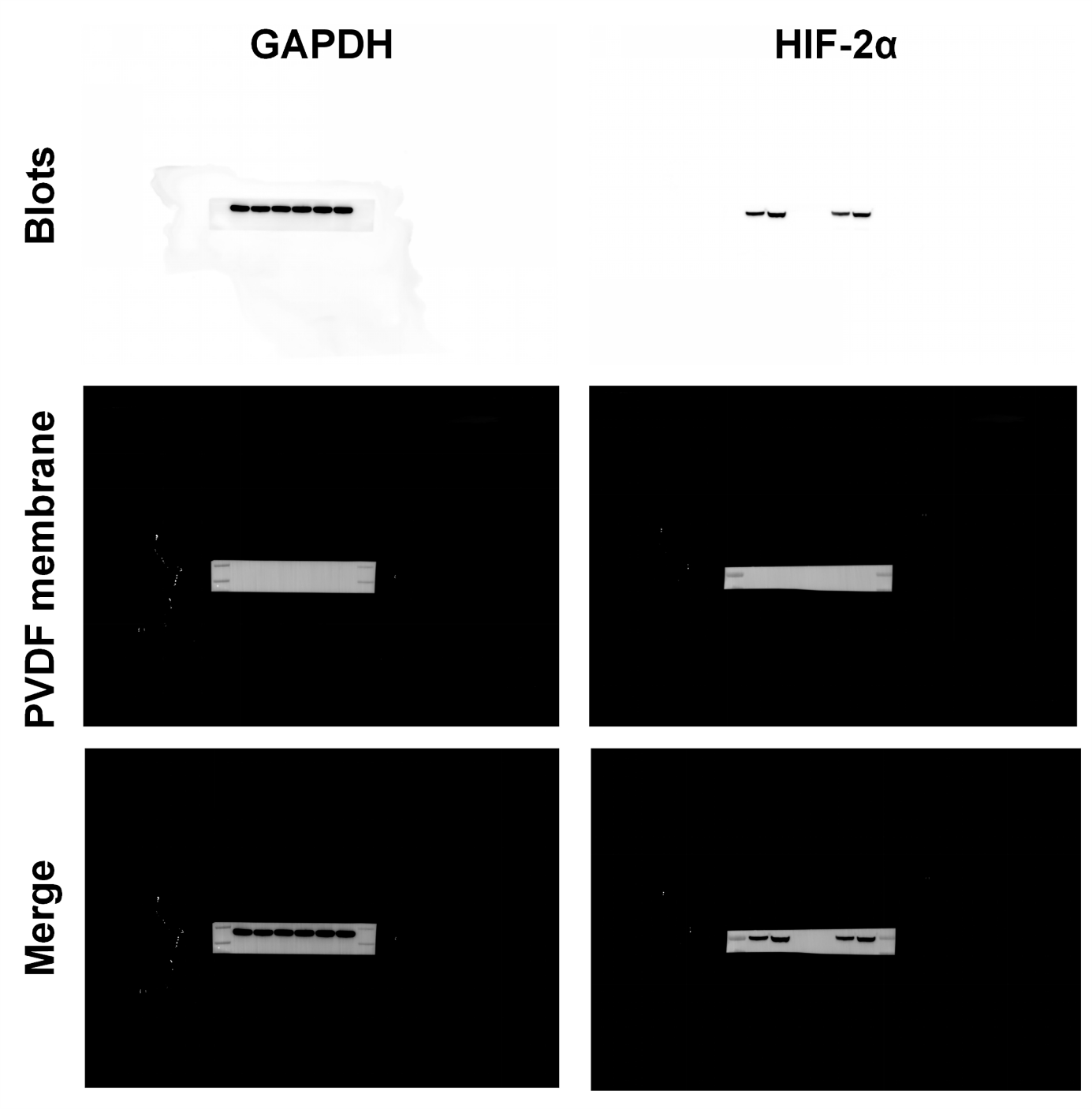


**Figure S8. Uncropped images of the Western blot in Figure S2A.**

**
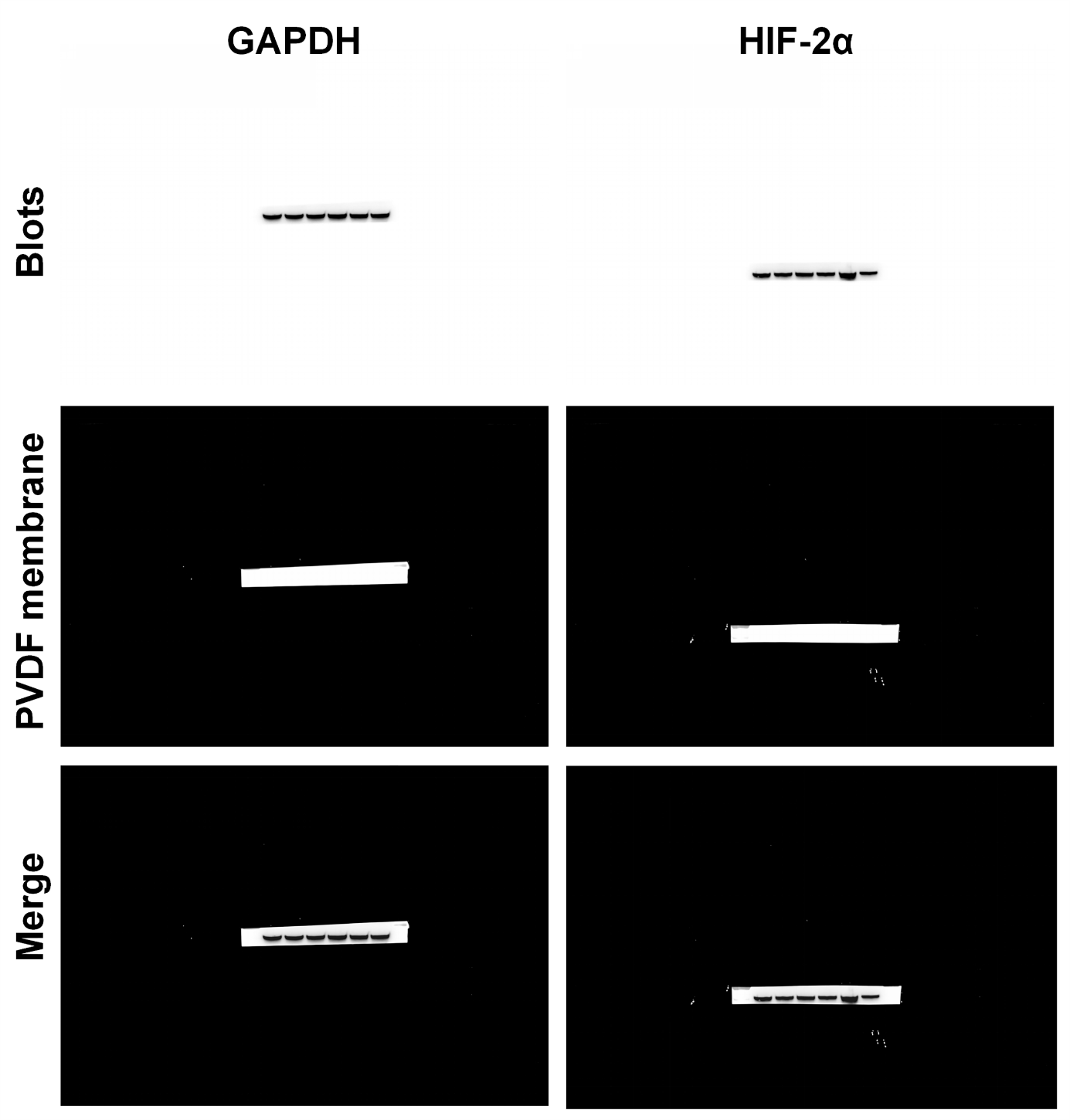
**

**Figure S9. Uncropped images of the Western blot in Figure 4A.**

**
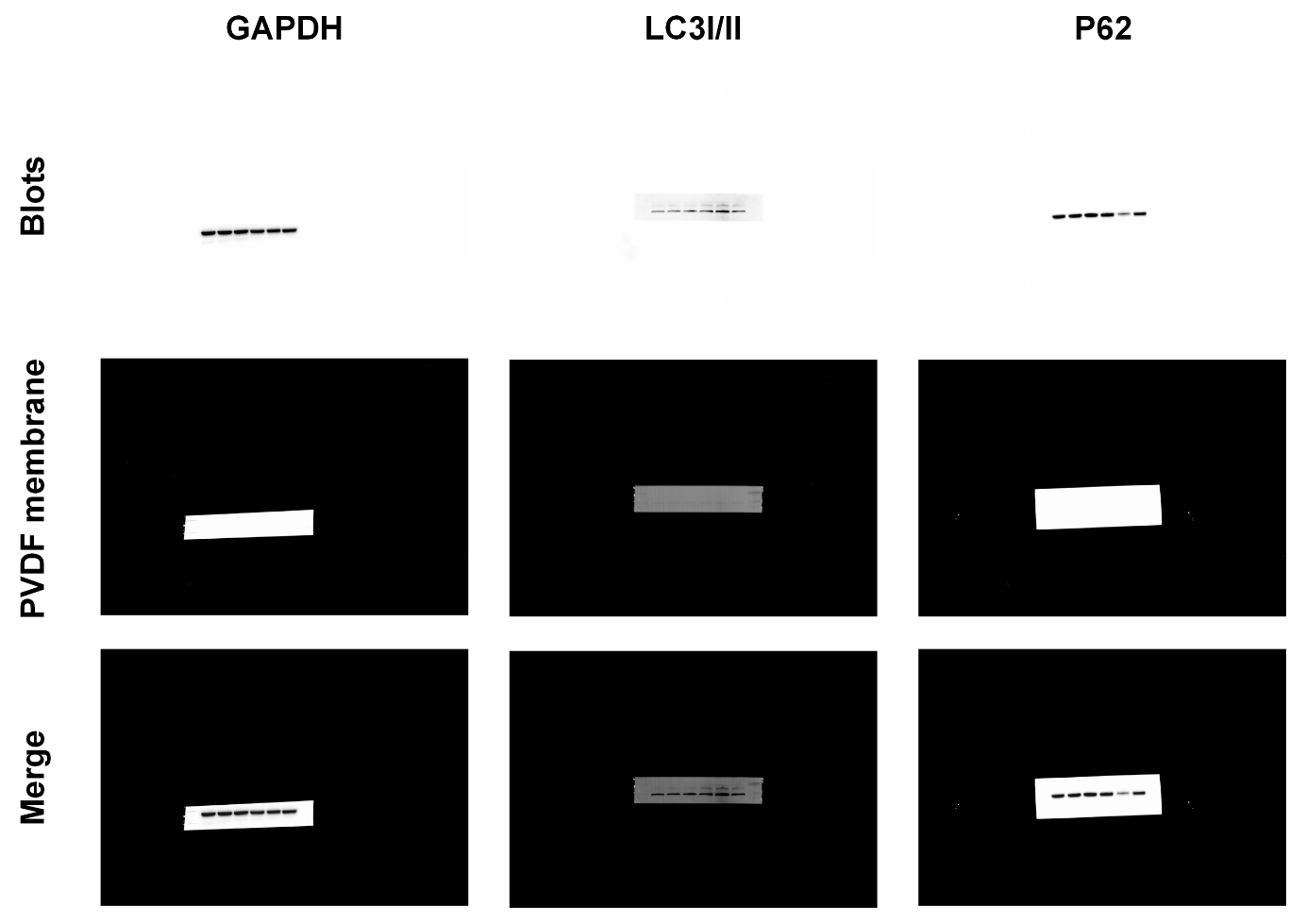
**

**Figure S10. Uncropped images of the Western blot in Figure 4C.**

**
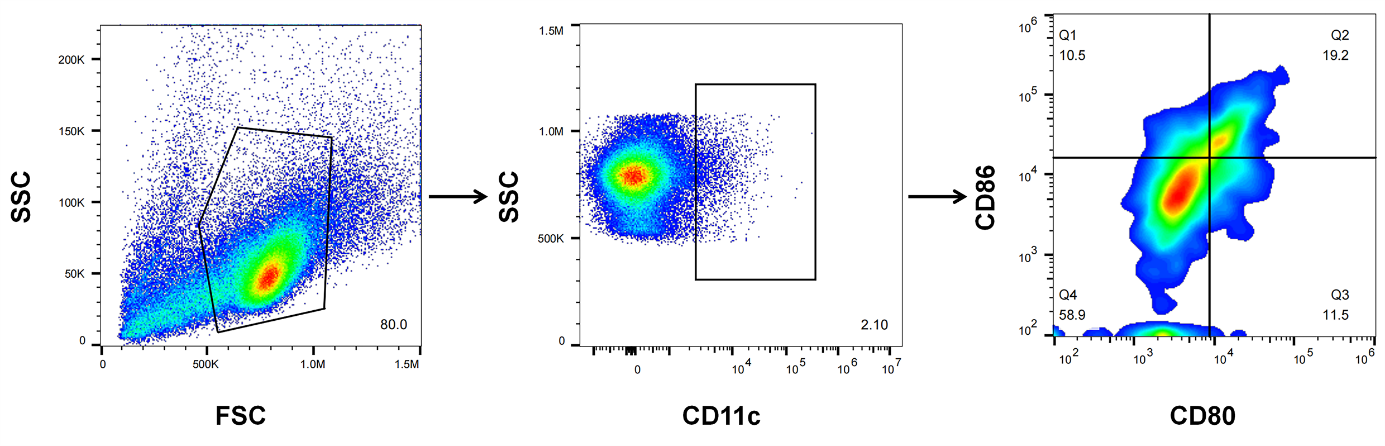
**

**Figure S11. The gating strategy of mature DCs in tumor-draining lymph nodes (TDLNs), wherein matured DCs were denoted as CD80^+^CD86^+^ cells.**

**
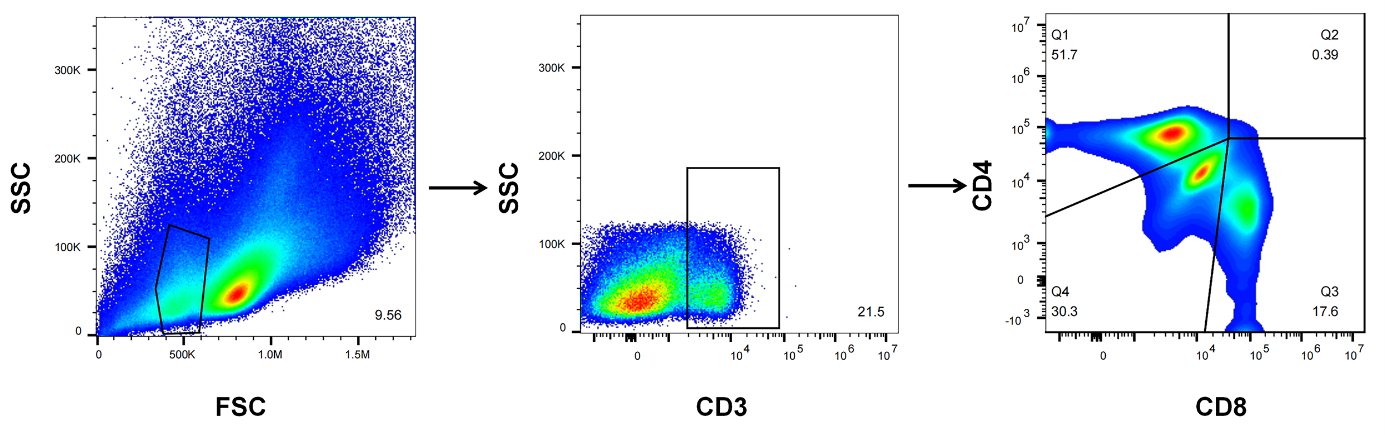
**

**Figure S12. The gating strategy of CD3^+^CD8^+^ T cells in spleens, which was denoted as the percentage of CD8^+^ T cells in the population of CD3^+^ T cells.**

**
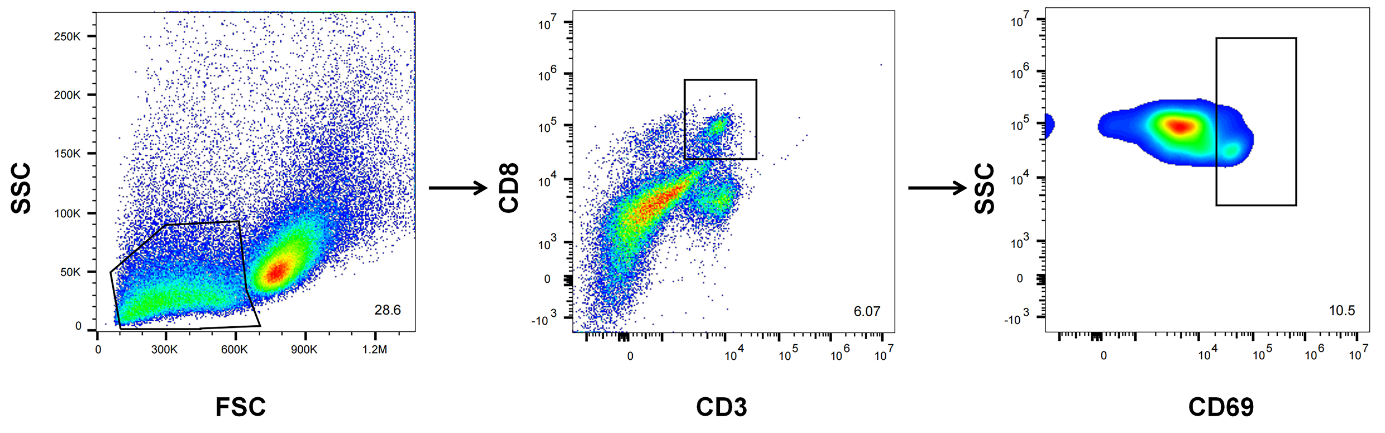
**

**Figure S13. The gating strategy of CD3^+^CD8^+^CD69^+^ T cells in spleens, which was denoted as the percentage of T cell activation.**

**
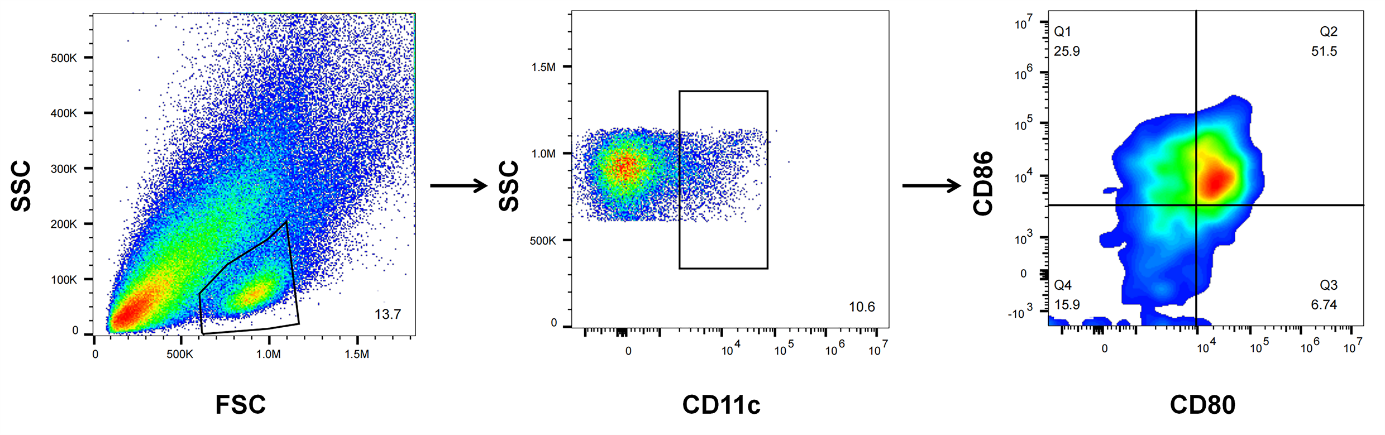
**

**Figure S14. The gating strategy of mature DCs in tumors, wherein matured DCs were denoted as CD80^+^CD86^+^ cells.**

**
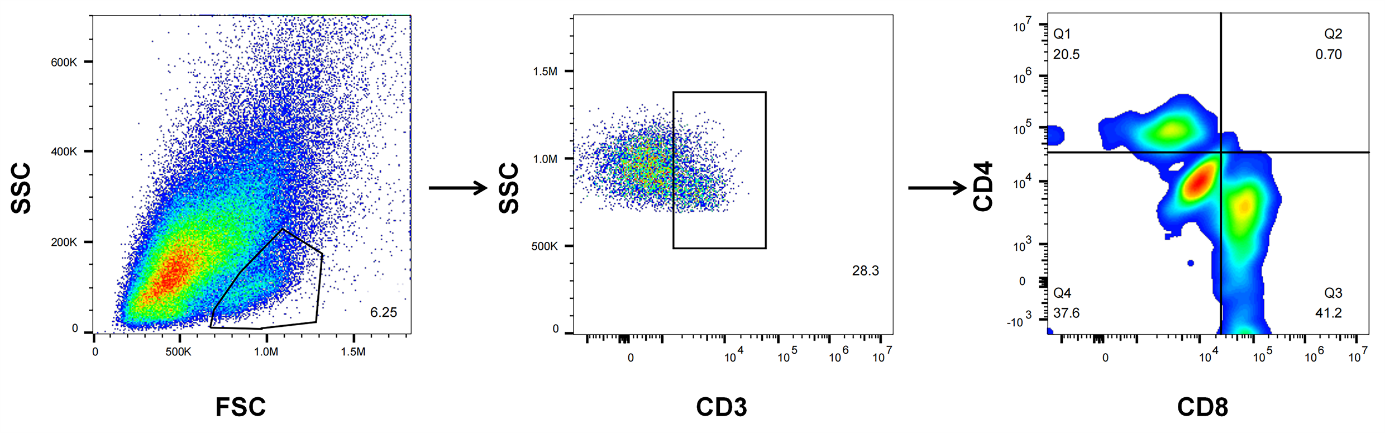
**

**Figure S15. The gating strategy of CD3^+^CD8^+^ T cells in tumors, which was denoted as the percentage of CD8^+^ T cells in the population of CD3^+^ T cells.**

**
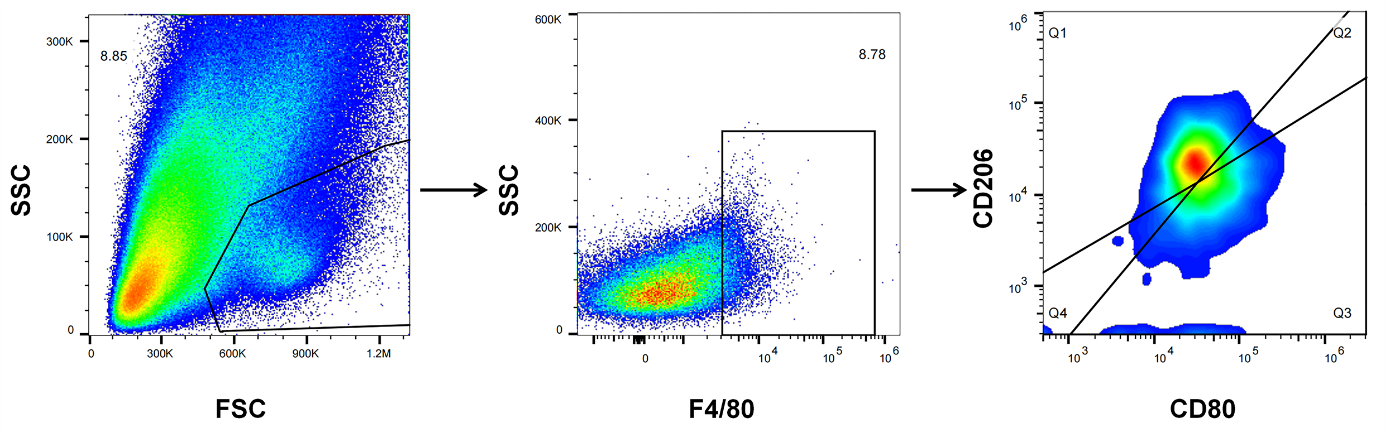
**

**Figure S16. The gating strategies of M1 and M2-phenotype macrophages in tumors. Macrophages were denoted as F4/80 positive cells, wherein M1-phenotype macrophages were F4/80^+^CD80^+^CD206^-^cells and M2-phenotype macrophages were F4/80^+^CD80^-^CD206^+^cells.**

**
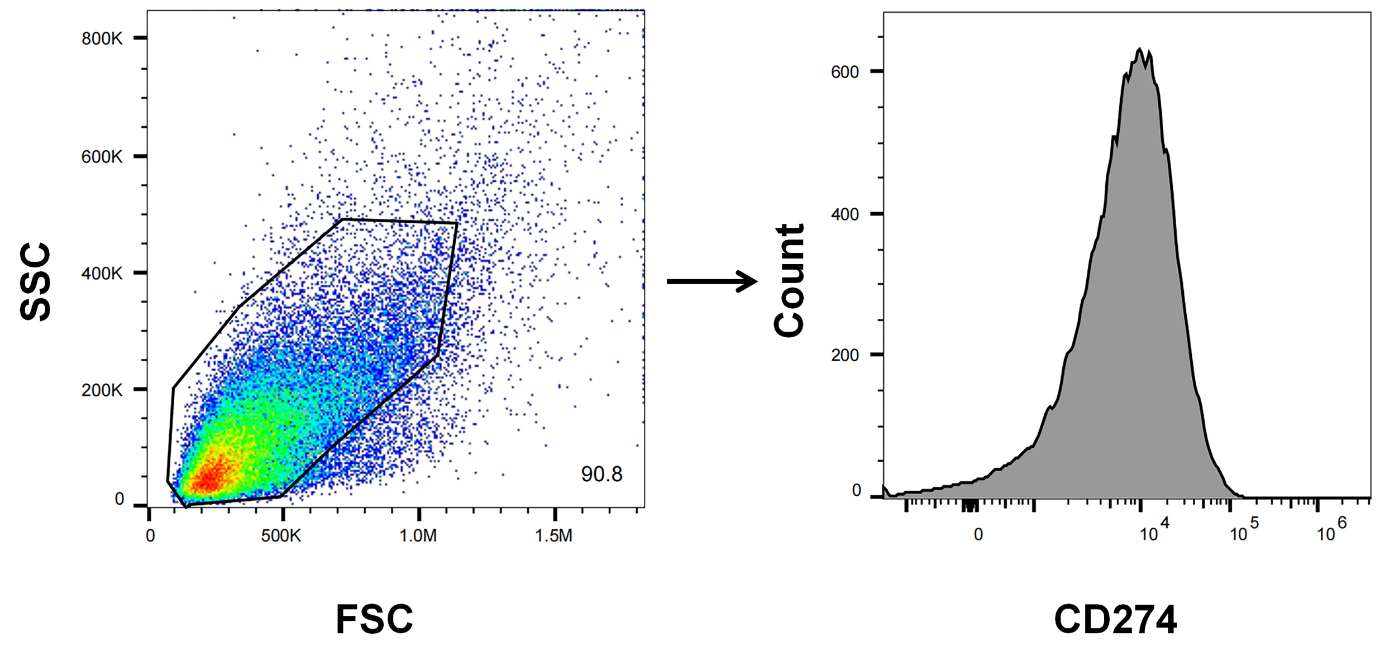
**

**Figure S17. The gating strategy of PD-L1 expression in tumors, wherein PD-L1 were denoted as CD274^+^ cells.**

**
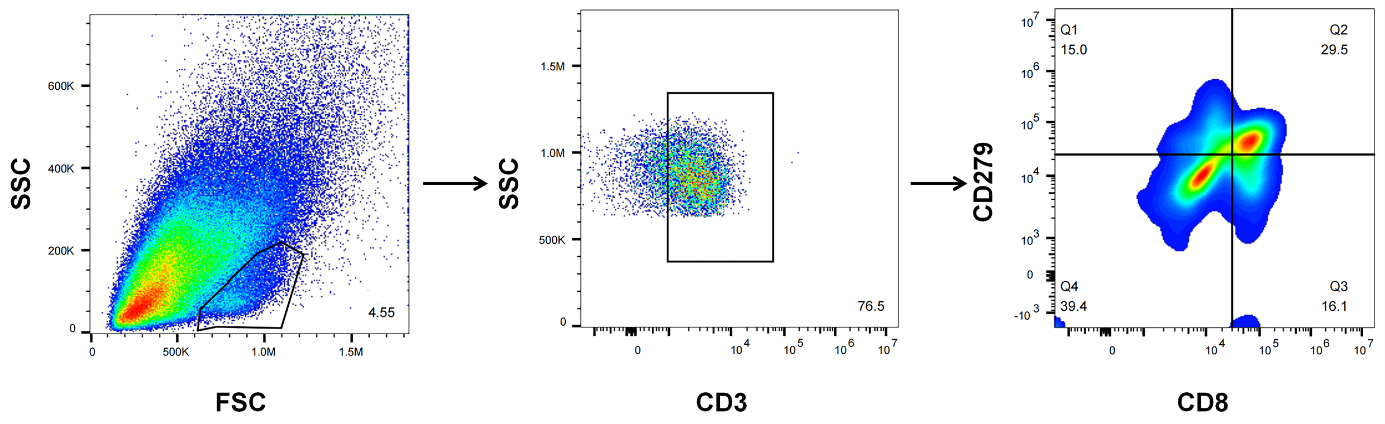
**

**Figure S18. The gating strategy of CD3^+^CD8^+^CD279^+^ T cells in tumors, which was denoted as the percentage of PD-1^+^ T cells.**
